# Benefits and Risks of Smallholder Livestock Production on Child Nutrition in Low- and Middle-Income Countries

**DOI:** 10.3389/fnut.2021.751686

**Published:** 2021-10-27

**Authors:** Dehao Chen, Karah Mechlowitz, Xiaolong Li, Nancy Schaefer, Arie H. Havelaar, Sarah L. McKune

**Affiliations:** ^1^Department of Environmental and Global Health, College of Public Health and Health Professions, University of Florida, Gainesville, FL, United States; ^2^Emerging Pathogens Institute, University of Florida, Gainesville, FL, United States; ^3^Department of Social and Behavioral Sciences, College of Public Health and Health Professions, University of Florida, Gainesville, FL, United States; ^4^Health Science Center Libraries, University of Florida, Gainesville, FL, United States; ^5^Department of Animal Sciences, Institute of Food and Agricultural Sciences, University of Florida, Gainesville, FL, United States; ^6^Institute for Sustainable Food Systems, University of Florida, Gainesville, FL, United States; ^7^Center for African Studies, University of Florida, Gainesville, FL, United States

**Keywords:** child nutrition, livestock, gut health, enteric pathogens, risk factors, animal feces, WaSH (water sanitation and hygiene), environmental enteric dysfunction (EED)

## Abstract

Livestock production may improve nutritional outcomes of pregnant women and children by increasing household income, availability of nutrient-dense foods, and women's empowerment. Nevertheless, the relationship is complex, and the nutritional status of children may be impaired by presence of or proximity to livestock and their pathogens. In this paper, we review the benefits and risks of livestock production on child nutrition. Evidence supports the nutritional benefits of livestock farming through income, production, and women's empowerment. Increasing animal source food consumption requires a combination of efforts, including improved animal management so that herd size is adequate to meet household income needs and consumption and addressing sociocultural and gendered norms. Evidence supports the inclusion of behavior change communication strategies into livestock production interventions to facilitate the sustainability of nutritional benefits over time, particularly interventions that engage women and foster dimensions of women's empowerment. In evaluating the risks of livestock production, evidence indicates that a broad range of enteric pathogens may chronically infect the intestines of children and, in combination with dietary deficits, may cause environmental enteric dysfunction (EED), a chronic inflammation of the gut. Some of the most important pathogens associated with EED are zoonotic in nature with livestock as their main reservoir. Very few studies have aimed to understand which livestock species contribute most to colonization with these pathogens, or how to reduce transmission. Control at the point of exposure has been investigated in a few studies, but much less effort has been spent on improving animal husbandry practices, which may have additional benefits. There is an urgent need for dedicated and long-term research to understand which livestock species contribute most to exposure of young children to zoonotic enteric pathogens, to test the potential of a wide range of intervention methods, to assess their effectiveness in randomized trials, and to assure their broad adaptation and sustainability. This review highlights the benefits and risks of livestock production on child nutrition. In addition to identifying research gaps, findings support inclusion of poor gut health as an immediate determinant of child undernutrition, expanding the established UNICEF framework which includes only inadequate diet and disease.

## Introduction

Globally, undernutrition underlies nearly half of mortality of children under five (CU5) (1). Stunting and wasting, indicators of chronic and recent undernutrition, affected more than 149 and 45 million CU5, respectively, in 2020 (2). Stunted (defined as length/height (L/HAZ)-for-age Z scores < −2) children are more likely to encounter recurrent illness, experience lower vaccine effectiveness, and have poorer intellectual and emotional development, leading to lower financial attainment in the later stages of life and higher morbidity and mortality (3–5). Wasting (defined as weight-for-length/height (WL/H) Z scores < −2) is more acute and directly increases the risk of mortality in CU5 (2). Though debate remains about the reversibility of undernutrition, evidence indicates that children who are undernourished are more likely to become obese or overweight later in life (6–8), thus creating a dual lifetime burden from malnutrition.

Most current understanding of child nutrition is based on the well-established United Nations Children's Fund (UNICEF) framework, which identifies the determinants of undernutrition in children, consisting of *immediate causes* (inadequate dietary intake and diseases), *underlying causes* (household food security, inadequate care and feeding practices, and unhealthy household environment and inadequate health services), and *basic causes* (social, cultural, economic, livelihood, etc.) (9). In low- and middle-income countries (LMIC), agriculture has been linked to improved child nutrition outcomes through three primary pathways: food production, income, and women's empowerment (10). These pathways can be explored within the UNICEF framework to understand how small-scale (smallholder) livestock production, which promotes the potential to produce nutrient dense, high value animal source foods (ASF) and enhance socio-economic well-being (11, 12), can affect child nutritional outcomes. While livestock *production* is frequently mentioned in nutrition-sensitive agriculture as a mechanism to promote ASF consumption, literature examining the relationship between livestock production and ASF consumption is limited, complex, and the results are inconsistent (13). Most directly, livestock production may produce ASF that is consumed by the household, including children. The production pathway's influence on child nutrition varies widely, including by food item, market engagement, and other contextual factors (14). Socioeconomic status, cultural practices that limit or restrict ASF consumption, the capacity and/or opportunity for storage and preservation of ASF, community and household-level gender dynamics, and intra-household resource allocation are all factors that can affect individual ASF consumption (15). Many households engage in *selling* livestock or livestock products for income, which complicates an understanding of how livestock production may affect diets. Livestock production and productivity may require a certain threshold before households consume ASF produced on-farm—if at all. However, households that sell ASF produced on-farm may still benefit from improved diet composition and overall well-being given the increased purchasing power of the household (16–19). In addition to production and income, *women's empowerment* serves as a pathway from agriculture to improved child nutrition. Compared to other land or financial assets, women can access and control livestock as an agricultural asset (20). Women's ownership or co-ownership of livestock has been associated with improved nutritional outcomes compared to male ownership (21). Livestock production can help women build and secure their ownership over assets, providing a source of regular income that can contribute to a pathway out of poverty (22). Women who earn more, have greater control over income and other financial resources, and have more decision-making power are more likely to ensure better household health and nutrition (23–26).

Nevertheless, connections between livestock ownership and child nutrition are complex. Although small-scale livestock production presents an opportunity for increased ASF production/consumption to halt child undernutrition in LMIC (27, 28), a 2018 systematic review revealed no congruent relationship between ASF consumption and the alleviation of common undernutrition outcomes, including stunting (29).

Furthermore, although many observational studies significantly associate improved family-level water, sanitation, and hygiene (WaSH) with improved child linear growth and lessened burden of environmental enteric dysfunction (EED), a key mediator of growth faltering (30–32), these significant effects did not persist in landmark randomized controlled trials (WASH-Benefits Bangladesh & Kenya, SHINE) (33–36). Researchers suggested that unmeasured household risk factors could have confounded the beneficial effect of WaSH interventions, and called for a “transformative WaSH” approach to curtail fecal contamination at the household level in future research (33). Notwithstanding, a recent study applying this transformative approach found its intervention, without addressing environmental contaminations (substandard food hygiene, animal feces, soil), was associated with easing of intestinal epithelial damage but had no effect on gut inflammation, and the protective effect did not strengthen linear growth (37). Negative outcomes from these trials applying traditional and improved WaSH measures to improve child nutrition underscore the need to incorporate a more holistic view of WaSH, such that the role of livestock management and exposure to animal feces are considered in future rural intervention programs (38).

Why must livestock production be considered a risk? Livestock serve as reservoirs of many zoonotic pathogens, which affect both human and animal health globally (28). These zoonotic diseases not only have the potential to reduce production of labor and commodities by the farm animals, but they can also threaten food security, food safety, and the health and livelihoods of the farming households (39). More than 60% of emerging pathogens and over half of the identified human pathogen species are zoonotic; this pathogen group has been attributed to over $20 billion direct loss and more than $200 billion indirect loss globally (40–43). Though emerging zoonotic pathogens with pandemic capacity have received extensive research attention, considerably less attention has been given to endemic livestock-related zoonotic enteric pathogens sourced from their feces and typically transmitted through the fecal-oral pathways characterized by the well-known “F-diagram” (41, 44). A 2019 study estimated LMIC have a much higher burden of enteric pathogens of foodborne disease from ASF (45), which are commonly produced in low-resource non-intensive smallholding farming systems, typically with fewer cleaning, disinfection, and biosecurity practices (11, 28). Thus, smallholder households in LMIC may be more susceptible to the adverse effects of a high burden of zoonotic enteric pathogens driven directly or indirectly by exposure to animal excreta (46). Enteric pathogen infection may not only cause acute diseases such as diarrhea, but, more importantly, may cause subclinical enteric infection (47). These asymptomatic infections have been hypothesized to result in EED, a subclinical gastrointestinal disorder occurring in low-resource settings in LMIC (48). With the slow advancement of curtailing child undernutrition globally and the awareness that this crisis cannot be solely ascribed to poor dietary intake and diarrheal illnesses, EED has been suggested to be the primary mediator between exposure to enteric pathogens in environments and undernutrition (49–53).

**Figure 1:**
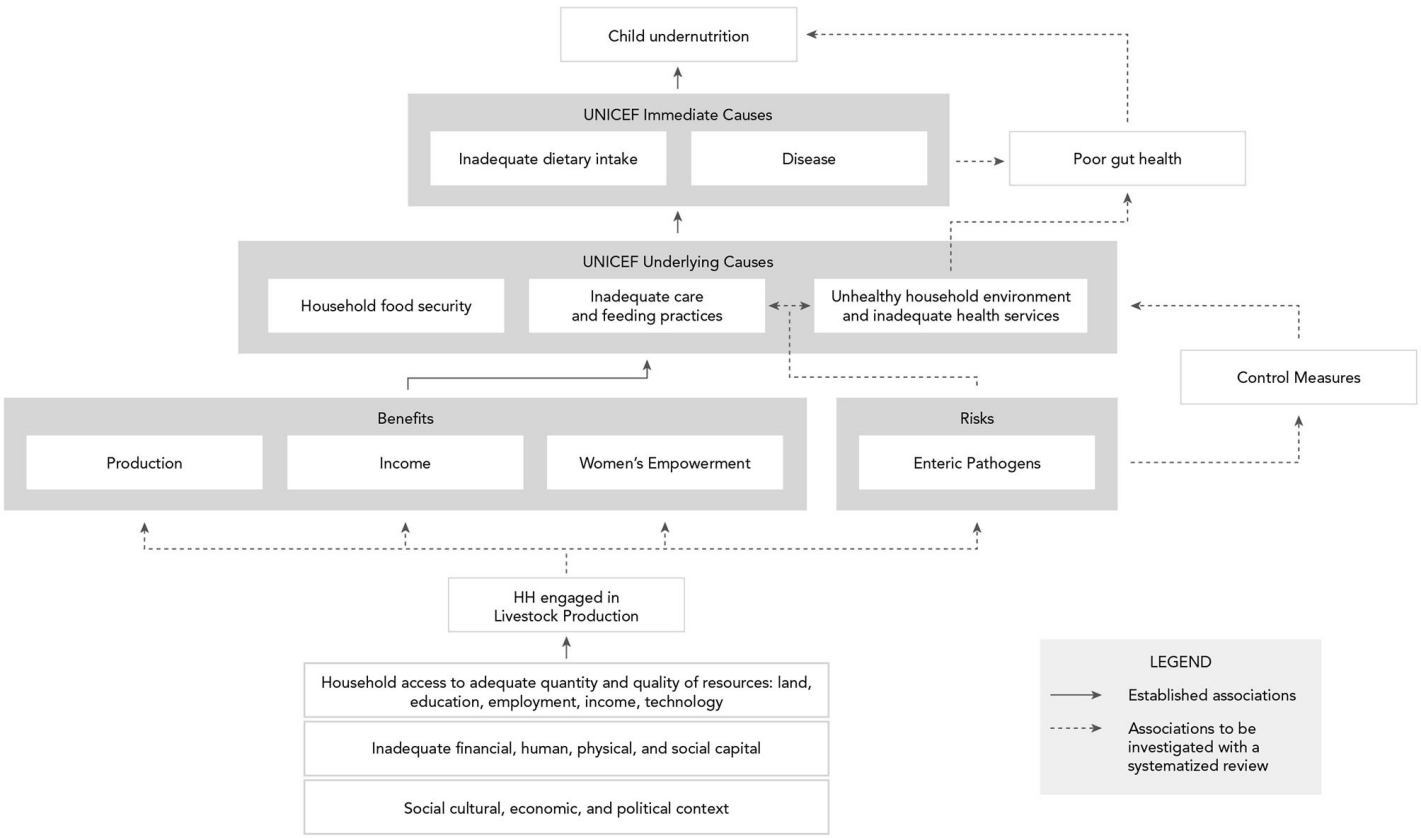
Conceptual framework of benefits and risks of smallholder livestock production on child nutrition in low- and middle-income countries.

In Figure 1, the agriculture to nutrition pathways previously described are mapped onto the UNICEF framework to demonstrate how smallholder livestock production (SLP) among rural households can affect child nutrition through benefits of established pathways. In addition, the risks of enteric pathogens' negative impact on child nutrition through inadequate feeding, care practices, and contamination of the household environment have been added, along with their potential, without control measures in place, to undermine child growth through impaired gut health. Therein emerges our hypothesis, that *the nutritional benefits of ASF in smallholder families may be negated by children's exposure to zoonotic enteric pathogens from animal feces, in the absence of adequate sanitation and biosecurity protocols*. Although discipline-based systematic reviews have been independently conducted on the relationships between ASF consumption and child growth indicators (29) and between exposure to animal feces and certain human health outcomes (46), no risk-benefit analysis has sought to jointly characterize the benefits and risks of ASF farming on child nutrition in LMIC. Here, using an integrated and multidisciplinary approach, we conduct a systematized review to address this knowledge gap. Specific research questions (see Table 1) address the associations depicted with dashed arrows in Figure 1. Established associations depicted by solid arrows in Figure 1 will be included in the synthesis based on existing reviews.

**Table 1:** Research questions on benefits and risks of smallholder livestock production on child nutrition in low- and middle- income countries (LMIC).

**Benefits of smallholder livestock production**	**Corresponding result sections**
1. How does smallholder livestock production affect children's consumption of ASF produced on-farm?	Production
2. How does income generate from smallholder livestock production affect children's diets?	Income
3. How does engaging in smallholder livestock and poultry production impact women's empowerment?	Empowerment
**Risks of smallholder livestock production**	
1. Which enteric pathogens are associated with EED, or undernutrition outcomes in children under 5 years of age?	Risks of smallholder livestock production
2. What environmental and socioeconomic risk factors and transmission pathways contribute to children's exposure to zoonotic enteric pathogens in smallholder households?	Risks factors of exposure to or infection with zoonotic enteric pathogens associated with smallholder livestock production
3. What attribution and control methods are available to track the source of and reduce children's exposure to zoonotic enteric pathogens in smallholder households?	Attribution of zoonotic enteric pathogen infections in LMIC; Control measures of zoonotic enteric pathogen infections in LMIC

As such, our work aims to synthesize the existing literature and evidence base on the role of livestock production on the WaSH landscape in relation to nutritional outcomes and pinpoint relevant research gaps and priorities through a systematized review.

## Methods

Between March and June 2021 we performed systematized searches of the literature for the research questions shown in Table 1. We searched PubMed, Agricola, CABI, and Ebsco's Academic Search Premier, EconLit, CINAHL, and Women's Studies International databases for the benefit aspects and Web of Science Core Collection, PubMed, Embase, and CABI for the risk aspects using MeSH terms (PubMed) and truncated and phrase-searched keywords in the title or abstract. See Appendix for details on search strategies by research question, eligibility criteria, and the number of articles identified and screened. We included English-language full text materials published between January 2000 and June 2021. Duplicate results were removed, and we then screened title-abstract and then full-text with support of the Covidence software (54). We went on to include relevant articles from personal libraries, as well as references noted in full-text screening of articles generated. We extracted data from full text of included studies in spreadsheets then synthesized and summarized results. Given research team composition and expertise, cross fertilization of results and findings occurred at each stage of data extraction, synthesis and summary. Discussion section and final conclusions were developed collaboratively to ensure integration of findings.

## Results

### Benefits of Smallholder Livestock Production

This section reviews the benefits of SLP. It is structured around production, income, and women's empowerment (Supplementary Table 1). Women's empowerment is broken down into the major domains of women's empowerment identified in the literature.

#### Production

Of the 145 records included, 24 reported results from interventions that investigated the impact of a livestock intervention on ASF consumption and/or dietary intake of children and/or households. Of the nine studies that measured child ASF consumption, a significant increase in child ASF consumption from baseline to endline was reported in one study (54); significant increases in child ASF consumption among intervention compared to control households were reported in five studies (55–59); two studies found no significant differences (60, 61); and one study did not report significance (62). Of the 4 studies that reported child dietary intake data, significant increases in child dietary diversity among intervention compared to control households were reported in two studies (57, 61), a marginally statistically significant greater increase in children's minimum dietary diversity among intervention compared to control households was reported in one study (63), and no significant difference in child intake of foods groups among intervention compared to control households was reported in one study (60).

Of the livestock intervention studies, 19 reported dietary data at the household level, 12 of which reported ASF consumption data at the household level. Of these 12 studies, significant increases in household ASF consumption from baseline to endline were reported in two studies (64, 65); significant increases in household ASF consumption among intervention compared to control households were reported in four studies (58, 59, 66, 67); no significant increases in household ASF consumption among intervention compared to control households were reported in three studies (68–70); and significance was not reported in three studies (61, 71, 72). Of the 19 studies that measured household dietary diversity, significant differences were reported in three studies (58, 61, 73); a marginally statistically significant greater increase among intervention compared to control households was reported in one study (63); and no significant differences were reported in three studies (67, 74, 75). Furthermore, of these 19 studies, one study reported a significantly lower percentage of food insecure households among intervention compared to control households (76); one study reported a significant improvement in food security among intervention compared to control households (77); one study reported a significant increase in household consumption of green, leafy vegetables and yellow/orange fruit among intervention compared to control households (61); and one study found no significant differences in household energy intake among intervention compared to control households (54).

Educational or behavior change communication (BCC) components that promote optimal care and feeding practices, health seeking behaviors, and/or knowledge were common in livestock production interventions, with 13 interventions including a BCC component. Of these 13 studies, five reported child ASF consumption data. Four of these five studies reported significantly greater increase in child ASF consumption among intervention households with the educational or BCC component compared to control households (55–58), while one study reported no significant difference in child ASF consumption among intervention compared to control households (61). Of these 13 studies, five reported household ASF consumption data. Two of five studies reported significant increases in household ASF consumption in intervention households from baseline to endline (64, 65); two studies reported significant increases in household ASF consumption among intervention households with the educational or BCC component compared to control households (58, 67); and one study did not report significance for the differences in household ASF consumption observed (72).

Of the 13 studies with BCC interventions, six reported household dietary diversity data. Of these six studies, three studies reported significant increases in household dietary diversity among intervention households with the educational or BCC component compared to control households (58, 61, 67); one study reported marginally statistically significant increase in household dietary diversity scores among intervention compared to control households (63); and two studies reported no significant differences (74, 75). Furthermore, results from one intervention that included a technical assistance component reported stronger program effects on income from livestock and higher consumption from own production for participants who received technical assistance compared to those who did not (77). The authors also reported an increase in program impacts with treatment exposure and length.

Of the 145 records included, 70 were observational studies investigating livestock production and dietary intake. Of these, five studies reported significant results for livestock production and increased child ASF intake (21, 78–81), five studies reported significant results for livestock production and increased household ASF intake (82–86), and two studies found no significance between livestock production and child ASF intake (87, 88). Regarding dietary intake, three studies reported significant results between livestock production and improved household dietary intake, including household food availability and household food calories (89), food consumption score (90), and household food consumption (91); remaining studies did not measure the association between livestock production and dietary intake. Among the observational studies, 40 described smallholder farmers' objectives for producing livestock. Of these, 23 reported ASF consumption from livestock production as a primary reason for livestock production, eight studies reported ASF consumption from production as not one of the primary reasons, and two studies did not assess ranking of objectives.

#### Income

Of the 145 records included, 65 records investigated income and livestock production. Of these, 13 studies reported results from interventions that examined the effect of livestock production interventions on household income, nine of which reported that the interventions significantly increased household income from livestock sales (57–61, 64, 69, 73, 77), two of which reported no significant effects of the intervention on household income (67, 70), and two of which did not report significance values (62, 68). Furthermore, four of the 13 studies reported that increased income from livestock sales through program participation led to improved household nutritional outcomes through enhanced purchasing power for food, which can include ASF (58, 60, 73, 77).

Of the 65 records investigating income and livestock production, 52 were observational studies, four of which measured the association between livestock production and income. All four found positive results: one study reported a significant association between cow ownership and increased dairy income (86), one study reported significantly higher household incomes among households with livestock (92), one study reported that camel milk was a significant contributor to household income (93), and one study reported that livestock ownership was a determinant of household income (94). Three studies measured associations between income from livestock and nutritional outcomes. Of these, one study reported that income from livestock sales was significantly higher among households with adequate food consumption compared to households with poor and intermediate food consumption (91), one study reported that livestock income significantly increased food expenditure (95), and one study reported no effect of livestock income on household dietary diversity (96).

#### Empowerment

Of the 145 records included, 42 studies investigated livestock production and women's empowerment. Of these studies, eight studies were intervention studies and 33 were observational studies. The women's empowerment in livestock index (WELI), developed in 2015, is a quantitative assessment of the empowerment of women in agriculture, specifically focused on livestock production. It was developed in response to the women's empowerment in agriculture index (WEAI), which mainly focuses on the empowerment of women in agriculture in general, with less attention given specifically to livestock production (97). Given the gendered challenges and opportunities involved in livestock production, the WELI was developed to better address the measurement of women's empowerment in livestock production (20). The WELI identifies six dimensions of empowerment in livestock production: decisions about production, access to and control over resources, control and use of income, access to and control of opportunities, workload and control over time, and decisions related to nutrition (20). Though many studies investigated more than one dimension of the WELI, and most did not name or use the WELI, the results from observational studies are organized based on the WELI dimensions of empowerment. The search terms for this review reflect our overarching goal to understand the benefits of livestock production on child nutrition. As such, this section is not a complete literature review of the WELI domains, but rather a way of categorizing results of the search for this review.

Nine of the 33 observational studies investigated women's empowerment in the context of decisions about production. Four studies discussed women's decision-making and role in small-ruminant production and reported that women tend to be more responsible for small ruminant activities, mainly poultry production (98–103), while men tend to have more responsibility over large ruminant production, such as cattle or camel (99, 101, 104). One study pointed to the head of household's importance in decision-making ability over livestock production, discussing how women in women-headed households were more likely to retain control over livestock production compared to women in men-headed households (105). Though women engaged in SLP can contribute significantly economically to the household, their decision-making is often constrained, depending on the type of livestock, intensification of the production system, sociocultural context, and the type of decision being made (16, 22, 99, 102). For example, women may be able to make decisions about livestock inputs or care strategies but not about the sale or slaughter of livestock and/or the use of livestock assets (99).

Twenty-two observational studies discussed women's empowerment in livestock in the context of having access to and control over resources. Larger livestock and income from larger livestock tend to be more under men's control (106), while smaller-scale livestock activities tend to be more under the control of women (101, 104). Poultry production, in particular, was the most widely cited production system under the control of women (99, 100, 103, 107–109). Increasing women's control over production and resources can facilitate better access to ASF for women and children (110).

Small animals, including poultry, are often the first animals sold to meet household needs, such as education or medical expenses (99, 111–113). However, one study reported that the decisions about the end use of high-value livestock (such as cattle) may involve a more joint-based decision-making approach due to such livestock's high economic and social value (99), but this remains to be further explored. While livestock production—particularly of small animals—is more readily accessible and controlled by women compared to other land or financial assets (20), the type of livestock owned and/or controlled by women can be culturally-and location-specific (22). The type of “rights” women may have over livestock production are heterogenous and can be divided into categories and sub-categories of rights such as resource access, right to withdraw or use products, and decision-making rights (22). “Ownership” of livestock does not necessarily entitle women to make decisions about it or about income generated from the livestock (99).

Eleven observational studies discussed women's empowerment in the context of control and use of income. Some evidence suggests that women may have more control over income from small ruminant, poultry, and dairy products (99, 101, 114, 115). Some evidence suggests that as a livestock production activity becomes more lucrative, the dynamics of control between men and women change, with more control shifting to men (107, 116, 117). Similarly, a transition toward commercialization, larger-scale production, or formalized markets may shift control and income toward men (104).

Only one observational study discussed the effect of group membership and women's empowerment in livestock production, noting that women in groups had more control over income from dairy products that those that were not in groups (118).

Eleven observational studies discussed livestock production and workload and control over time. The effect of livestock production on women's workload and control over time was mixed, with some evidence indicating a greater workload and labor demand among women engaged in livestock production (99, 102, 107, 119). Because women in smallholder farming households tend to be primarily responsible for domestic duties, such as caring for children and preparing food, livestock production activities are often performed in addition to their daily responsibilities (120). While some parts of production may be shared among household members, women are often more responsible for the day-to-day management of production (particularly for poultry production), including feeding, watering, and caring for livestock (103). However, three studies report differentiated impacts of livestock production on workload and time burden depending on the type of livestock (121), how intensive the production system is (120), and if the household is man vs. woman-headed (105). The implications of increasing livestock productivity on women's workload and well-being can be complex and unpredictable (99). Even when they invest time and labor into livestock production, women may not have control over the sale and/or resulting revenue (99, 122, 123).

Women's decision-making power related to the use of livestock derived ASF for household consumption varies. Even when women participate in livestock-related activities and generate ASF, they may not have control over the use of ASF from livestock production (123). Milk and poultry production are areas where women may be the main decision-makers and have more control over the use of ASF within the household (101, 114, 124, 125). Additionally, if a household generates a surplus of an ASF, then women may be given control over the surplus to use or sell (102).

The allocation of ASF within the household is not always equal, with individual access to ASF depending on myriad factors, including gender, age, and type of ASF (99). Dairy products, for example, may be more equally allocated within the household, with children often being a primary recipient, compared to meat (21, 99). Furthermore, co-owned or female-owned livestock has been found to be significantly associated with child ASF intake when compared to male ownership (21).

Having reviewed the literature from observational studies, the following section presents findings from intervention studies. The results of livestock production interventions on women's empowerment showed mixed effects, as some were significant (55, 75, 77, 126) and others non-significant (57, 59, 124, 127). Significant effects of livestock production interventions included increased women's decision-making about livestock products (55); improved overall empowerment score (75); increased women's empowerment as measured by the Women's Empowerment in Agriculture Index (WEAI) (77); and increased scores in social capital, asset access, financial empowerment, and agriculture empowerment domains (126). Regarding intervention effects on ASF consumption, significant increases in child ASF consumption were associated with interventions in three studies (55, 57, 59), while the remaining studies did not measure child ASF consumption. Significant increases in household milk consumption (59), child dietary diversity (57), and household food security (77) between intervention and control groups were reported. A non-significant difference in household dietary diversity between the intervention group and the control group was reported in one study (75). Incorporation of a BCC or technical assistance component as part of the livestock production interventions was common. A mix of significant increases (55, 75, 77, 126) and non-significant (57, 127) differences of livestock production interventions with BCC components on women's empowerment was reported.

Having mapped out the benefits of livestock production through the three primary pathways of production, income, and empowerment, it is important to acknowledge that in the absence of these protective elements, there is an increased risk of poor nutrition outcomes. Livestock production is a key resource for many smallholder farming households in LMIC, particularly for women. When these beneficial factors are not present, the pathway from livestock production to nutrition can counterfactually pose risks to child nutrition, in tandem with exposure to enteric pathogens.

### Risks of Smallholder Livestock Production

#### Enteric Pathogens as Determinants of EED or Undernutrition in Children Under Five

In this section, we review evidence from studies on enteric pathogens associated with EED and/or undernutrition outcomes in CU5 and characterize their reservoirs and risk factors for infection. We identified 12 and 38 articles that found enteric pathogens statistically associated with EED and undernutrition, respectively. Despite being the gold standard of diagnosing EED (128), endoscopic biopsy is often not feasible in epidemiologic studies. Thus, in the reviewed studies, EED was characterized by biomarkers of different impairments to gut function as discussed in Tickell et al. (34), Rogawski and Guerrant (129).

Table 2 summarizes study results of pathogens significantly associated (*P* < 0.05) with increased levels of EED markers and/or undernutrition outcomes in CU5 and their reservoirs. Details regarding effect sizes of the associations between pathogen infections and the health endpoints are provided in Supplementary Tables 2, 3. Although this review is focused on zoonotic enteric pathogens, we include anthroponotic and sapronotic pathogens because of the limited level of speciation for some pathogens in the literature, and to offer a more comprehensive portrayal of enteric pathogens involved in the pathogenesis of EED and undernutrition (Table 2). Following WHO's definition, we classified the reservoir of infection as zoonotic (Z) if it is naturally transmissible from vertebrates to humans. We classified pathogens as anthroponotic (A) if the reservoir is exclusively human. Anthroponotic pathogens originating from a single zoonotic spillover event but lacking epidemiologic evidence on sustained zoonotic transmission (e.g., rotavirus, *Cryptosporidium hominis* subtype IfA12G2) were characterized as anthroponotic (180, 181). Pathogens mainly replicating in abiotic environments (water, soil, food) were categorized as sapronotic (S) (182). In the reviewed literature, the pathogens were characterized taxonomically at different levels of detail by their biological group, genus, species, and subtype. Lacking sufficient taxonomic data, we assigned pathogens to different reservoirs (e.g., A/Z) as appropriate.

**Table 2:** Enteric pathogens associated with increased risk of EED or undernutrition at the 95% confidence level.

					**EED**		**Undernutrition**	
**Pathogen group**	**Genus/common name**	**Species**	**Subtype**	Reservoir^*^(References)	**Affected gut function^&^**	**References**	**Outcome^@^**	**References**
Viruses	Adenovirus			A (130)	Gut inflammation	(131)	–HAZ	(132)
	Norovirus			A (133)	N/A	N/A	–HAZ	(132)
	Rotavirus			A (134)	N/A	N/A	–HAZ	(132)
Bacteria	*Aeromonas*	spp.		S (135)	Epithelial damage/repair	(131)	N/A	N/A
	*Campylobacter*	spp.		Z (136)	Epithelial damage/repair, gut inflammation, intestinal permeability	(131, 137, 138)	–LAZ, –weight	(138–140)
		*jejuni/coli*		Z (136)	N/A	N/A	–LAZ	(141, 142)
	*Escherichia*	*coli*	Enterotoxigenic	A (143)	Gut inflammation	(131, 137, 144)	WAZ < −2, –LAZ	(141, 145)
			Enteroaggregative	A (143)	Gut inflammation, epithelial damage/repair	(131, 146–148)	–LAZ	(139, 147)
			Enteroinvasive	A (143)	Gut inflammation, intestinal permeability	(131)	N/A	N/A
			Enteropathogenic	A (143)	Epithelial damage/repair	(131)	N/A	N/A
	*Helicobacter*	*pylori*		A (149)	Epithelial damage/repair	(150)	N/A	N/A
	*Plesiomonas*	*shigelloides*		S (151)	Gut inflammation	(131)	N/A	N/A
	*Salmonella*	spp.		A/Z (152)	Epithelial damage/repair	(131)	N/A	N/A
	*Shigella*	spp.		A (153)	Gut inflammation, intestinal permeability	(131)	–LAZ, –height	(131, 139, 154)
	*Yersinia*	*enterocolitica*		Z (155)	Gut inflammation	(131)	N/A	N/A
Protozoa	*Cryptosporidium*	spp.		A/Z (156)	Intestinal permeability, epithelial damage/repair	(131, 157)	–LAZ, –WAVZ, –LAVZ, WAZ < −2	(145, 158, 159)
		*hominis*		A (156)	N/A	N/A	LAZ < −3, HAZ < -2, WAZ < −2, WHZ < −2	(160, 161)^$^
		*parvum*		A/Z (162)	N/A	N/A	HAZ < −2, WAZ < −2, WHZ < −2	(163)
	*Entamoeba*	*histolytica*		A (164)	N/A	N/A	–WAZ, WAZ < −2	(145, 165)
	*Giardia*	spp.		A/Z (166)	Gut inflammation, epithelial damage/repair, intestinal permeability, microbial translocation	(131, 137, 148, 167)	–LAZ, –WAZ,	(139, 167, 168)
		*lamblia*		A/Z (166)	Intestinal permeability	(169)	HAZ < −2, WHZ < −2, –LAZ, –WAZ, –WHZ, MUAC <12.5 cm, –LAD	(159, 170–174)
Geohelminths				A^∧^	N/A	N/A	LAZ < −2, –LAZ, –LAD	(159, 175)
	*Ascaris*	*lumbricoides*		A (176)	N/A	N/A	–HAZ	(172)
	Hookworm			A (177)	N/A	N/A	–LAZ	(165)
	*Trichuris*	spp.		A (176)	Gut inflammation, epithelial damage/repair	(148)	N/A	N/A
		*trichiura*		A (176)	N/A	N/A	HAZ < −2	(178)

* *A, anthroponotic; Z, zoonotic; S, sapronotic; A/Z, anthroponotic/zoonotic.*

& *Markers in original literature measuring the gut functions: myeloperoxidase, neopterin, calprotectin, lactoferrin—gut inflammation; regenerating protein 1β, α-1-antitrypsin, intestinal fatty acid binding protein—epithelial damage/repair; lactulose:mannitol—intestinal permeability; anti-flic immunoglobulin A—microbial translocation.*

@ *Undernutrition outcomes associated with the infections included binary undernutrition indicators based on anthropometric Z-scores (i.e., severe stunting, stunting, wasting, underweight) or common thresholds (i.e., MUAC <12.5 cm), and slow gains in the Z-scores (i.e., –L/HAZ, –WAZ, –WHZ) and weight and height velocities (i.e., –WAVZ, –LAVZ) (179) or crude anthropometric measures (i.e., -weight, -height), and length-for-age difference (-LAD). MUAC, mid-upper arm circumference; L/HAZ, length/height-for-age Z score; WAZ, weight-for-age Z score; WHZ, weight-for-height Z score; WAVZ and LAVZ, weight and length velocities; LAD, length-for-age difference.*

$ *Cryptosporidium hominis was the dominant species in the affected populations.*

∧ *Geohelminths consisted of Ascaris spp., Trichuris spp., hookworm*.

Four studies associated enteroaggregative *E. coli* (EAEC) with the increased biomarkers of gut inflammation [lactoferrin, neopterin (NEO), myeloperoxidase (MPO)] and epithelial damage/repair [α-1-antitrypsin (AAT)] (131, 146–148), and two studies found increased levels of this pathogen to be associated with linear growth faltering (139, 147). Three studies found gut inflammation (measured by calprotectin (CAL) and MPO) was positively associated with enterotoxigenic *E. coli* (ETEC) infection (131, 137, 144). In contrast, a negative association was found in one study between ETEC and the inflammation marker NEO (131). Two studies demonstrated ETEC increased the risk of underweight and growth faltering (141, 145). Three studies reported a directionally heterogeneous association between *Campylobacter* spp. and markers of gut inflammation (131, 137, 138). All studies found a positive association with MPO. Again, a negative association was found in two studies with NEO (131, 138). As found by 2 and 1 studies respectively, *Campylobacter* spp. also elevated AAT, the marker of epithelial damage/repair and the lactulose and mannitol (L:M) ratio indicating increased intestinal permeability (131, 137, 138). Regarding undernutrition, 3 studies demonstrated *Campylobacter* infection increased the risk of reduced weight gain and growth faltering (138–140). Diagnostic methods in the MAL-ED study included an immunoassay targeting all (thermotolerant and non-thermotolerant) *Campylobacter* species (138) and a molecular assay targeting the (thermotolerant) species *C*. jejuni and *C*. coli (139). The effect size (measured as LAZ difference between children in the upper 10-percentile of Campylobacter burden vs. those in the lower 10-percentile) for *Campylobacter* spp. was approximately twice as high as for *C. jejuni/coli* (−0.33 vs. −0.17), suggesting one or more non-thermotolerant species are strongly associated with linear growth faltering. *Shigell*a spp. were found to increase the markers of gut inflammation (MPO) and intestinal permeability (L:M) by one study and linear growth faltering and slow gain of length by two studies (131, 139, 154).

Kosek et al. found that enteroinvasive *E. coli* and *Plesiomonas shigelloides* were both associated with elevation of MPO, with the former also associated with increased L:M ratio (131). In contrast, decreased levels of biomarkers of epithelial damage or repair (AAT) were observed for enteropathogenic *E. coli* (EPEC) and the genera *Salmonella* and *Aeromonas*. *Yersinia enterocolitica* was associated with elevated MPO and decreased NEO, while both are markers of gut inflammation (131). *Helicobacter pylori* was found by one study in association with biomarkers of epithelial damage or repair (AAT, Reg1β) but with opposite directions of effects (150).

The enteric protozoa *Cryptosporidium* spp. and *Giardia* spp. were also associated with EED and undernutrition endpoints. At the genus level, *Cryptosporidium* was found in two studies to increase biomarkers of intestinal permeability (L:M) and epithelial damage/repair [intestinal fatty acid binding protein (I-FABP)] (131, 157), while 3 studies revealed the pathogen increased the risk of not only the common undernutrition endpoints of the (binarized) Z-scores of length and weight but slow velocity gains (measured by length and weight velocity Z-scores) of the two measures (145, 158, 159). Four studies associated *Giardia* spp. with markers of gut inflammation (MPO, NEO), epithelial damage/repair [AAT, regenerating protein 1β (Reg 1β)], intestinal permeability (L:M), and microbial translocation [anti-flic immunoglobulin A (Flic IgA)]. However, the effect(s) on the inflammation endpoint, when indicated by MPO were directionally reversed in two studies and were negative when solely measured by NEO in one study (131, 137, 148, 167). Other than the common undernutrition outcomes, at the species level, infection with *G. lamblia* also contributed to slow gain of length-for-age difference (-*LAD*) (159). Two studies associated *Entamoeba histolytica* with underweight and slow gain of weight-for-age Z score (WAZ) (145, 165).

Geohelminths consist of multiple anthroponotic parasites. When evaluated as a group, it was associated with linear growth faltering, stunting, and slow gain of LAD (159, 175), while hookworm was associated by one study with growth faltering (165). *Trichuris* spp., a genus of geohelminths, was found by one study to increase a composite EED score of MPO, NEO, and AAT (148). *T. trichiura* was positively associated with stunting (178).

Two studies associated adenovirus with elevated MPO and growth faltering (131, 132). Diarrheal illness by rotavirus and norovirus was found to increase the risk of growth faltering (132).

Several studies confirmed the strong associations between diarrheal viral and bacterial infections with reduced weight and height gain and growth faltering as suggested by the original UNICEF framework. More recently, studies have associated asymptomatic infections of *Campylobacter* spp. and EAEC and Giardia with similar undernutrition endpoints (139, 139, 140). Importantly, one study of *Campylobacter* infections in Peru found that asymptomatic infection had a larger effect size (−65.5 vs. −43.9 g) on weight gain vs. symptomatic infections (140).

More generally, extensive infection with enteric pathogens, including those that were not delineated in Table 2, might also be associated with EED or undernutrition (131). A 2021 study in Zambia associated cumulative burden of enteric pathogens (consisting of Shiga toxin producing *E. coli* (STEC) and others) in fecal samples with elevated concentrations of I-FABP, a marker of epithelial damage or repair (157). A 2019 study in an asymptomatic child cohort in Pakistan found, among the 40 enteric pathogens studied, presence of any enteric pathogen in a stool sample was positively correlated with flagellin IgA and Reg 1b, which are EED biomarkers of increased microbial translocation and epithelial damage/repair, respectively. Multiple infections of enteric pathogens, measured by pathogen counts, were negatively associated with growth faltering at 18 months of age (137). A 2020 study in Ethiopia suggested the group infected with at least one of seven detected intestinal parasites in stool specimens had significantly higher stunting prevalence than those that were not (183).

Furthermore, other than enteric pathogens, virtually any bacteria at high concentrations in the small bowel could cause EED (184). When normal composition of gut microbiota is disrupted, even commensal microorganisms, which are related to small intestinal bacterial overgrowth (SIBO) (185), are associated with EED and stunting. A Bangladesh study revealed fecal Reg1β and CAL were higher in SIBO-positive children (185). A study in sub-Saharan Africa associated overgrowth of bacteria belonging to the oropharyngeal taxa with stunting (186). A 2020 study suggested the elevating and reducing quantities of Proteobacteria and *Prevotella* spp. were associated with poor linear growth in Bangladesh, accordingly (187).

#### Animal Models of Enteric Pathogen Infections, Environmental Enteric Dysfunction, and Undernutrition

Given the observed epidemiological associations between (asymptomatic) infections with particular pathogens, including bacteria (e.g., *E. coli* pathotypes, *Shigella* spp., *Campylobacter* spp.) and intestinal protozoa (e.g., *Giardia* spp. and *Cryptosporidium* spp.), we investigated evidence from animal studies supportive of causal associations and found they have been reviewed previously (188, 189). One review that assessed mouse models of EED suggested that a combination of nutrient (protein, zinc) deficient diets and (a)symptomatic gut infections by enteric pathogens including *Cryptosporidium parvum* and *Campylobacter jejuni* could induce EED or EED-like conditions and/or growth restriction (188). Both reviews underlined that EED and growth restriction are complex and heterogeneous processes and advocated further advancements of animal studies through investigating the gut health and nutrition outcomes jointly (188, 189). A mouse model found that although undernutrition triggered by deficiencies of micronutrients and/or macronutrients could impede growth, the nutritional deficiencies alone were not sufficient to induce enteropathy. The combination of infections and nutrient deficient diets impaired the gut barrier and caused uncontrolled inflammatory response triggering growth failure (190). Peculiarly, a mouse model suggested respiratory infections by influenza viruses led to intestinal damage through a complicated immunological mechanism, indicating this respiratory pathogen might be involved in the etiology of EED (191, 192). Nonetheless, how these results can be extrapolated to humans remains uncertain (193).

#### Undernutrition as Risk Factor of Enteric Pathogen Infections

Undernutrition is an established risk factor for diarrheal and other infectious diseases (194). We found 7 articles identifying undernutrition [i.e., growth faltering, (severe) stunting, wasting, underweight] as significant risk factors of enteric pathogen infections, as measured by a positive stool sample that could be either symptomatic (i.e., diarrheal) or asymptomatic (195–201). While wasting was a risk factor of more aggravated diarrheal symptoms related to rotavirus infections (195), stunting and severe stunting were significant risk factors of diarrheal/non-diarrheal *Cryptosporidium* and *Aeromonas* infections, respectively (196–199). A 2020 study suggested wasted infants had higher odds of having a stool sample positive for *Campylobacter* spp. (200).

### Risks Factors of Exposure to or Infection With Zoonotic Enteric Pathogens Associated With Smallholder Livestock Production

We identified 27 eligible studies investigating risk factors associated with direct/indirect exposure to zoonotic enteric pathogens associated with EED and undernutrition in CU5 in smallholder settings (Table 2).

The level of pathogen specification was limited in many eligible studies, as in the previous section. Four studies defined the pathogens at the genus level (e.g., *Salmonella, Cryptosporidium* spp.) (202–205), and 8 studies characterized pathogens with general biological groups only [i.e., enteric protozoa infection (206, 207), intestinal parasite infection (208–212), enteric pathogen infection (213)]. We therefore defined pathogens as zoonotic (Z) and as zoonotic and/or anthroponotic (A/Z) as in section Enteric pathogens as determinants of EED or undernutrition in children under five.

Household risk factors directly/indirectly contributing to children's exposure to and/or infection of zoonotic enteric pathogens are summarized by different transmission pathways (i.e., animal contact, foodborne, waterborne, person-to-person, and environmental) used by the US Centers for Disease Control and Prevention (CDC) for source attribution (214). Socio-demographic factors were designated according to CDC's social-ecological model (215). We classified factors related to WaSH into categories using the ladders of WHO/UNICEF's joint monitoring program (216–218).

#### Risks Through the Animal Contact Pathway

Risk factors classified as animal contact pathway are summarized in Table 3. One study found allowing random access to the farm was associated with increased level of *Salmonella* contamination on pen floors of livestock farm (202). Three studies positively correlated households' adjacency to livestock farms with fecal contamination (measured by *E. coli*) inside households and *Salmonella* on farm, as well as *Giardia lamblia* infection in CU5 (170, 202, 219). Four studies suggested owning livestock to be a risk factor for infection of CU5 or adults with enteric protozoa or *Campylobacter* spp. (200, 203, 206, 207). For families owning livestock, increasing livestock density in/around the households has been associated with visible indoor contamination of animal feces and infection by intestinal parasites (including *Giardia lamblia*) in household members (210, 220). A 2020 study associated presence of livestock feces (positive for *Campylobacter*) in/around households with infection of this pathogen in infants (200). Five studies found home slaughter or other direct contact with livestock were risk factors of zoonotic bacterial and parasitic pathogen infections in humans, in some cases accompanied by diarrhea (204, 211, 221–223). One of these five studies found that while animal contact was associated with *Cryptosporidium* infection at the genus level, further genotyping found *C. parvum* as the dominant species infecting livestock, whereas humans were mainly infected by *C. hominis* (222). Six studies suggested cohabitation with livestock was a risk factor of household contamination with livestock feces, intestinal parasite infection (containing *Giardia lamblia*), both symptomatic and asymptomatic infection with *Cryptosporidium* spp., *Campylobacter* spp., and other zoonotic enteric pathogens (e.g., STEC, *G. lamblia, Yersinia* spp.) in CU5 (200, 205, 209, 212, 213, 220). Other than exposure to livestock and their feces, a 2015 study associated frequent rodent sightings with the presence of non-typhoidal *Salmonella* in livestock, and *Cryptosporidium* infection in humans was positively correlated with presence of pet feces and scavengers in/around the households (221, 224, 225).

**Table 3:** Risk factors associated with the presence of zoonotic enteric pathogens in different endpoints (livestock, environment, human at all age range, and children under-five) through the animal contact pathway in smallholders in low- and middle- income countries (LMIC).

**Pathway**	**Risk factors**	Endpoints (pathogen reservoir)^&^^*^	**References**
Animal contact	Random access to farm by visitors	Environment: Contamination of *Salmonella* spp. (A/Z) on farm's pen floors	(202)
	Living in close proximity to livestock	Environment: fecal contamination in household, indicated by *E. coli* (A/Z); contamination of *Salmonella* spp. (A/Z) on farm	(202, 219)
		CU5: *Giardia lamblia* infection (A/Z)	(170)
	Livestock ownership	Human: Enteric protozoa (A/Z) infection; *Cryptosporidium* spp. infection (A/Z)	(203, 206, 207)
		CU5: *Campylobacter* spp. infection (Z)	(200)
	Increasing livestock density/quantity	Environment: visual presence of livestock feces in household	(220)
	inside household/at farm	Human: Intestinal parasite infection (A/Z)	(210)
	Presence of (pathogen-positive) livestock feces in/around households	CU5: *Campylobacter* spp. infection (Z)	(200)
	Direct contact with (diarrheal) livestock	Human: (symptomatic) *Cryptosporidium* spp. infection (A/Z); symptomatic *Campylobacter* spp. infection (Z)	(204, 221, 222)
	Home/informal slaughtering of livestock	Human: *Campylobacter jejuni* infection (Z), Intestinal parasite infection (A/Z)	(211, 223)
	Cohabiting with/Keeping livestock	Environment: presence of livestock feces in household	(220)
	inside household	Human: intestinal parasite infection (A/Z)	(209)
		CU5: (symptomatic) *Cryptosporidium* spp. infection (A/Z); (symptomatic) *Campylobacter* spp. infection (Z); infection of zoonotic enteric pathogens (Z)	(200, 205, 212, 213)
	Presence of pet feces in/around household	Human: symptomatic *Cryptosporidium* spp. infection (A/Z)	(221)
	Presence of rodent	Livestock: presence of non-typhoidal *Salmonella* (Z)	(224)
	Presence of scavengers	Human: *Cryptosporidium* spp. infection (A/Z)	(225)

& *Endpoints related to zoonotic enteric pathogen infection driven by the risk factors are characterized at the following levels: (1) Livestock: presence of zoonotic enteric pathogens in livestock; (2) Environment: presence of zoonotic enteric pathogens in the environment in/around a household (e.g., soil, water, food, fomites); (3) Human: (a)symptomatic zoonotic enteric pathogen infection in humans of all ages; (4) CU5 (children under five): (a)symptomatic zoonotic enteric pathogen infection in CU5.*

* *A, anthroponotic; Z, zoonotic; A/Z, anthroponotic/zoonotic*.

#### Risk Through Waterborne and Environmental Pathways

Seven studies reported risk factors through the waterborne and environmental pathways in smallholder settings (Table 4). One study found that using untreated water for cleaning was associated with higher level of fecal indicator *E.coli* in the household environment (219), and another study associated having poor access to safe drinking water with infection by *G. lamblia* and other intestinal parasites in humans (208). Two studies revealed that household crowding was associated with an elevated burden of infection with intestinal parasites, including *G. lamblia* (208, 212). *Two* studies found that improper disposal of garbage was significantly associated with increased risks of *Campylobacter* diarrhea in CU5 and *Cryptosporidium* infection in humans (205, 221). One study in Ethiopian households raising chickens associated floor samples contaminated by *Campylobacter* spp. with infection by these bacteria in infants (200).

**Table 4:** Risk factors associated with the presence of zoonotic enteric pathogens in different endpoints (environment, human at all age range, and children under-five) through the waterborne and environmental pathways in smallholders in LMIC.

**Pathways**	**Risk factors**	Endpoints (pathogen reservoir)^&^^*^	**References**
Waterborne	Use of untreated water for household cleaning activities	Environment: fecal indicator *E. coli* (A/Z) in households	(219)
	Poor access to safe drinking water	Human: intestinal parasite infection (A/Z)	(208)
Environmental	Household crowding	Human: intestinal parasite infection (A/Z)	(208, 212)
	Poor garbage disposal manner	Human: *Cryptosporidium* spp. infection (A/Z)	(221)
		CU5: symptomatic *Campylobacter* spp. infection (Z)	(205)
	Exposure to contaminated floor (pathogen-positive) in household	CU5: *Campylobacter* spp. infection (Z)	(200)

& *Endpoints related to zoonotic enteric pathogen infection driven by the risk factors are characterized at the following levels: (1) Environment: presence of zoonotic enteric pathogens in the environment in/around a household (e.g., soil, water, food, fomites); (2) Human: (a)symptomatic zoonotic enteric pathogen infection in humans of all ages; (3) CU5 (children under five): (a)symptomatic zoonotic enteric pathogen infection in CU5.*

* *A, anthroponotic; Z, zoonotic; A/Z, anthroponotic/zoonotic*.

#### Other Risk Factors

Five studies reported other risk factors associated with infections of *Cryptosporidium, Campylobacter* and intestinal parasites (including *G. lamblia*) in CU5 and other household members (Table 5). One study in Tanzania identified residing with infected people as a risk factor of *Cryptosporidium* spp. infection (181). While chimpanzees and livestock were present in the study area, concurrent infections with *C. hominis* subtype IfA12G2 were found in chimpanzees and humans, while no common *Cryptosporidium* spp. infecting livestock and humans were identified, suggesting the person-to-person transmission might potentially be initiated by the zoonotic spillover from the chimpanzees (181). A study in Ethiopia suggested an increased *Campylobacter* burden in CU5 was associated with current breastfeeding and the intake of ASF (including raw milk) (32). Similarly, a study in Cambodia found raw meat consumption to be a risk factor of *C. jejuni* infection in humans (223).

**Table 5:** Risk factors associated with the presences of zoonotic enteric pathogens in different endpoints (human at all age range, and children under-five) fallen under the social-demographic category or through the foodborne and person-to-person pathways in smallholders in LMIC.

**Pathways**	**Risk factors**	Endpoints (pathogen reservoir)^&^^*^	**References**
Person-to-person	Sharing residence with infected people	Human: *Cryptosporidium hominis* infection (A) in a setting where animal reservoir (non-human primates) was present	(181)
Foodborne	Consumption of uncooked meat	Human: *Campylobacter jejuni* infection (Z)	(223)
	Current breastfeeding	CU5: *Campylobacter* spp. infection (Z)	(32)
	Animal source food consumption	CU5: *Campylobacter* spp. infection (Z)	(32)
**Levels in the social- ecological model (** **215** **)**			
Community	Residence in village	Human: *Cryptosporidium* spp. infection (A/Z)	(181, 222)
Relationship	Lower household SES	Human: Intestinal parasite (including *Giardia lamblia*) infection (A/Z)	(211)
	Lower level of adult education	Human: intestinal parasite (including *G. lamblia*) infection (A/Z)	(211)
Individual	Human: younger age	Human: *Campylobacter* spp. infection (Z)	(223)

& *Endpoints related to zoonotic enteric pathogen infection driven by the risk factors are characterized at the following levels: (1) Human: (a)symptomatic zoonotic enteric pathogen infection in humans of all ages; (2) CU5 (children under five): (a)symptomatic zoonotic enteric pathogen infection in CU5.*

* *A, anthroponotic; Z, zoonotic; A/Z, anthroponotic/zoonotic*.

Among the community-level socio-demographic risk factors, residing in villages was associated with *Cryptosporidium* spp. infection vs. living in city and camps in two studies (181, 222). Lower social-economic status at the household level was described by one study as a risk factor of intestinal parasites (including *G. lamblia*) infection, and this endpoint was correlated with a lower level of adult education (211). At the individual level, one study found younger age (<16 year old) increased the risk of *Campylobacter* infection in humans (223) (Table 5).

### Attribution of Zoonotic Enteric Pathogen Infections in LMIC

In LMIC's smallholder settings, risks of ASF production to child health are driven by exposure to zoonotic enteric pathogens from livestock and other animal reservoirs through different transmission pathways. Source attribution of zoonotic diseases can be conducted at the animal reservoir and vehicle (i.e., point of human exposure) levels (226). Established approaches for source attribution include microbiological approaches (e.g., microbial subtyping and comparative exposure assessment) and epidemiologic approaches as reviewed in section Risks factors of exposure to or infection with zoonotic enteric pathogens associated with smallholder livestock production. Here, we reviewed the potential and implementation of microbiological approaches to provide such quantification in LMIC. Applications of these approaches in high-income settings have been reviewed previously (226, 227).

Microbial subtyping methods are based on phenotypic or genotypic subtyping of isolates of pathogenic organisms from humans and different putative sources. Probabilistic models are used to attribute infection in humans to different (animal) sources. Non-living sources such as foods, waters and fomites can also be included, e.g., the application of multi-locus sequence typing (MLST) to attribute *Campylobacter jejuni* infecting human to livestock and environmental surface water sources in Europe (228).

Microbial Source Tracking (MST), as applied in WaSH studies, can also be classified as a microbiological approach. MST tracks the source of fecal contamination of water or other non-living transmission vehicles such as food or fomites to specific animal hosts by molecular detection of genetic markers particular to a host species occurring in commensal *Bacteroides* spp. (229). This method cannot be used to attribute infections in humans.

Comparative exposure assessment aims to characterize the relative significance of pre-determined vehicles of transmission through inferring the risk of human exposure to a pathogen of interest per vehicle. The assessment is conducted by evaluating levels of contamination in sources of interest and other transmission pathways, and exposure frequencies under each route, using statistical models.

We identified 15 studies using microbiological approaches implemented to quantify the relative contribution of different sources of human exposure to zoonotic enteric pathogens in LMIC −5 implemented the microbial subtyping approach which were found based on citation tracking from a previous review (230), 8 applied MST host-specific markers, and 2 utilized comparative exposure assessment.

#### Applications of Microbial Subtyping

All five studies utilizing the microbial subtyping approach aimed to attribute sources of non-typhoidal *Salmonella enterica* (NTS) infections in human populations (children or adults) in Africa. Subtyping methods included MLST, phage typing, pulsed-field gel electrophoresis (PFGE), AMR profiles, and plasmid typing (Table 6). Three of the five studies found that NTS recovered from children and adults in some African settings were originated from human reservoirs despite NTS is commonly considered to be zoonotic and close contacts between humans and animals were observed (231–233). In contrast, two other studies found PFGE patterns of NTS isolates from wildlife and poultry were matched to or clustered with those isolated from children and adults, suggesting zoonotic transmission (234, 235). On study further supported transmission from poultry by phage typing (235).

**Table 6:** Applications of microbial subtyping in low-resource settings.

**Approaches applied**	**Microorganisms attributed**	**Dominant sources identified**		**Countries**	**References**
		**Reservoirs (subtyping evidence)**	**Vehicles**		
Microbial subtyping—MLST^*^	NTS^*^	Humans (NTS genotypes from human & animals did not overlap)	–^#^	The Gambia	(231)
Microbial subtyping—PFGE^*^+ AMR^*^ profiles + plasmid typing		Humans (different PFGE patterns of NTS genomic DNA from human & poultry; 64.2% of NTS from human were resistant to antibiotics vs. all NTS from livestock and environment were susceptible)	–^%^	Kenya	(232)
		Humans (65.6% matching of NTS between people of contacts and index cases based on AMR profiles, and plasmid typing; matched PFGE patterns between index cases and people of contacts. 1.7% matching between NTS from environment sources and index cases)	–^%^		(233)
Microbial subtyping—PFGE^*^		Wildlife (Other than one wild species, all PFGE patterns of isolates from these animals were matched to an established pattern of human)	–^#^	South Africa	(234)
Microbial subtyping—PFGE^*^+ phage typing		Poultry (NTS isolates from poultry and humans owned the same phage type and their PFGE patterns were clustered)	–^#^	Burkina Faso	(235)

* *MLST, multi-locus sequence typing; PFGE, pulsed-field gel electrophoresis; NTS, non-typhoidal Salmonella; AMR, antimicrobial resistance.*

% *Environmental samples for source attribution were taken from this study to investigate potential vehicles, but no NTS were isolated from these samples or attribution patterns between isolates from the environment and humans were different.*

# *This study conducted source attribution only at the reservoir level and environmental samples were not taken*.

#### Applications of Host-Specific Microbial Source Tracking Markers

Among the eight studies implementing the MST approach (Table 7), two quantified the level of fecal contamination by animal or human hosts species in environmental samples. A study in Bangladesh found human fecal contamination affected nearly 80% of ponds in the study community (236). Another Bangladeshi study revealed a ruminant fecal marker was present in more than 30% of sampled produce from markets (238). Six studies statistically compared the presence of a host-specific marker between vehicles or combined MST with epidemiologic data. Three of these found levels of human specific *Bacteroides* spp. were significantly higher in sanitary wastewater, home environment, and wet soil (closed to a public latrine) than in stored drinking water, ponds and tube wells, and dry soil (237, 241, 242). A study in India found human and animal fecal markers were more often detected on mothers' and children's hands than in household stored drinking water (240). Another study in Bangladesh positively associated the animal marker on mothers' hands with levels of common enteric pathogen genes (i.e., *Giardia lamblia*, pathogenic *E. coli*) on these hands (239). Utilizing markers of three reservoir types (i.e., human, avian, dog), a study in Peru found household floors were more contaminated by the feces from all three species than tables, and wooden tables were more contaminated by the feces from the non-human species (243). More importantly, an avian marker was associated with the presence of *Campylobacter* on environmental surfaces (243). Presence of *Campylobacter* in household surfaces was associated with fecal contamination from poultry, indicated by an avian host-specific marker (243). Increased levels of human and animal markers in household were associated with the occurrence of diarrhea in children (241).

**Table 7:** Applications of host-specific microbial source tracking (MST) markers of fecal contamination in low-resource settings.

**Approaches applied**	**Microorganisms attributed**	**Primary sources identified**		**Countries**	**References**
		**Reservoirs, indicated by MST markers**	**Vehicles**		
Microbial source tracking (MST)	Commensal *Bacteroides* spp.	Humans	Pond water for bathing, fishing, hygiene use (public domain)	Bangladesh	(236)
		Humans	Sanitary wastewater in slums (public domain, comparing^*^ with stored drinking water)		(237)
		Ruminant livestock	Produce in markets (public domain)		(238)
		Animal (Pets & livestock)	Hands of mothers (household domain)		(239)
		Animal (Pets & livestock), humans	Hands of mothers and children (household domain, comparing^*^ with stored drinking water)	India	(240)
		Humans	Stored drinking water and humans' hands (household domain, comparing^*^ with tube wells and ponds)		(241)
		Humans	Visibly wet soil at the entrances to a public latrine (public domain, comparing^*^ with dry soil)	Mozambique	(242)
		Humans, poultry, dogs	Floors (household domain, comparing^*^ with tables for contaminations from human, poultry, and dog feces) Wood table surfaces (household domain, comparing^*^ with non-wood surfaces for contaminations from poultry and dog feces)	Peru	(243)

* *The comparison was made at the 95% confidence level*.

#### Comparative Exposure Assessment

Two eligible studies were belonged to the SaniPath analytical approach, based on collecting data from structured observations on child behavior, the concentration of generic *E. coli* in environmental samples and fitting the data into quantitative microbial risk assessment models to infer the relative risks of predetermined fecal exposure pathways (244, 245). Using parameters inferred from behavioral observation data, one study simulated daily behavioral sequences related to environmental fecal exposure of CU5 in an urban setting in Accra, Ghana. The simulation, based on the frequency of contact, indicated that younger children (<1 year) spent time predominantly off the ground, while the older children often entertained on the floor, suggesting that the floor could act as a key environmental source of exposure for the older CU5 group (246). The simulation also indicated that activities reducing fecal exposure, such as washing hands before eating, occurred infrequently (246). An exposure model combined the behavioral sequence model with a hierarchical model simulating *E. coli* concentrations in household environmental samples (245). The model suggested that food most substantially contributed (more than 99%) to fecal exposure, while hands likely acted as the key mediator between the mouthing of *E. coli* and environmental sources in the environments (245).

### Control Measures of Zoonotic Enteric Pathogen Infections in LMIC

The classic “F-diagram” depicts multiple pathways through which fecal microbes can be transmitted from human feces through contaminated water, soil, arthropod vectors, food and direct contact with contaminated environment to ingestion by healthy people (44). In this review, we discuss intervention methods for reducing the exposure to animal feces based on the adapted F-diagram referring to Penakalapati et al. (46) which extended the classic F-diagram by including animal feces into the diagram. To select appropriate intervention methods, it is important to quantify the relative weights of different (animal) reservoirs and pathways in terms of contribution to the transmission of pathogens. As discussed in section Attribution of zoonotic enteric pathogen infections in LMIC, very few studies have aimed to attribute infections with zoonotic enteric pathogens to animal reservoirs and even fewer have aimed to quantify exposure pathways. A total of 29 records were included after screening (Table 8).

**Table 8:** Control measures to prevent exposure to animal feces in LMIC.

**Category**	**Major pathway**	**Targeted arrow**	**Intervention**	**Major findings**	**References**
Animal waste management	Waterborne/ environmental	Animal feces –> fluids, fields	Biogas digesters	A low-cost plastic type digester in Cameroon considerably decreased the mean counts of coliform and *E. coli* in the slurries of chicken feces to 138 and 87 CFU/mL, respectively, after a five-week retention time in the digester	(247)
				The concentration of *Enterococcus spp*., *E. coli* and spores of *Cl. Perfringens* in pig slurries was reduced by 1–2 log CFU/mL after the treatment of biogas digesters in Vietnamese pig farms	(248)
				Biogas digesters with various designs can reduce the concentration of *Enterococci, E. coli*, and coliforms by 1 ~ 3.5 log_10_ CFU/mL in the manure	(249)
			Composting	The concentration of *E. coli* was reduced from 4 log_10_ CFU/g to below the detection limit in composted pig manure added 2% urea within 2 weeks. The counts of total coliforms decreased to 0.72 log10 CFU/g at day 45	(250)
Corralling domestic small animal	Animal contact	Animal feces –> fingers	Corralling free-range chicken	Corralling didn't fully achieve the goal of separating children from poultry as children in the household persistently played with corrals and with chickens in the corrals	(251)
				A significant higher (two-fold) incidence of *Campylobacter*-related diarrhea in children living in the corral group than in the control group	(252)
				The participant households in the corral group were revisited 10 years after the intervention, and majority of them (92.3%) had a positive change in their attitudes toward corral use	(253)
			Hutching small animals inside home	Participants reported that the three-level animal hatches constructed in kitchen and living space prevented rabbits and guinea pigs from defecating throughout the house which thus prevented children from eating animal feces on the floor	(254)
Baby WaSH	Animal contact/ environmental	Animal feces –> fingers, fields –> future victim	Provision of play-yard/play pen	The WaSH intervention package including providing plastic manufactured play-yards showed non-statistically significant effects on reducing both enteric infections and pathogen-attributable diarrhea caused by individual pathogens	(36, 255)
				The community-built play-yards (play-yards made from local materials) protected the young children from ingesting soil and livestock feces	(256)
			Provision of plastic playmats	Locally sourced plastic playmats were provided to caregivers in a pilot study, and they reported that use of playmats reduced the mouthing of dirt by children	(254)
Milk hygiene	Foodborne	Animal feces –> food, food –> future victim	Boiling or heating raw milk	The application of pasteurization in raw milk can cause the pathogen's probability of surviving to be reduced by a factor of 10^6^, and, consequently, destroy all vegetative microbes in the milk	(257, 258)
			Natural fermentation	The reduced pH along with the release of antimicrobial compounds by fermenting bacteria synergistically inhibit the growth of pathogenic microbes in the milk	(257)
			Smoking the inner surface of milk-handling containers	The mean microbial load in the smoked containers was reduced from 5.99 to 4.64 log_10_ cfu/cm^2^ for total viable count (TVC), from 5.07 to 4.00 log_10_ cfu/cm^2^ for total coliform count (TCC), and from 4.81 to 3.75 log_10_ cfu/cm^2^ for LAB	(259)
				The qualitative study conducted in Ethiopia identified the smoking of milk-handling containers as one of potential risk mitigation practices in the pastoral communities	(260)
Complementary food hygiene	Foodborne	Animal feces –> food, animal feces –> fomites, fomites –> food, fingers –> food, fomites –> future victim	Cleaning and disinfecting baby feeding bottles	Rinsing baby bottles with soapy water followed by tap water can reduce the load of fecal bacteria including EPEC and *Salmonella spp*. by 3.1 ~ 3.7 log_10_ CFU/mL	(261)
				The trial of improved practices showed that caregivers preferred the protocol of brushing the bottle with dish detergent for 30 s after every use than boiling the bottle for several minutes daily	(262)
			Intervention package of critical food hygiene behaviors	Six critical behaviors were identified and targeted: (1) handwashing before cooking; (2) handwashing before feeding; (3) washing cooking utensils with safe water and soap and drying them on a clean and elevated surface; (4) proper and safe storage of cooked food and utensils; (5) reheating food before feeding; (6) boiling drinking water. The results suggested a varying degree of increase in targeted complementary food hygiene behaviors	(263–269)
				Food samples collected 3 weeks after mother taking the training showed a significant reduction in the thermotolerant coliform (TTC) contamination levels which were reduced below 10 TTC/g for most samples cooled after cooking or reheated after storage	(270, 271)
				Besides the increased adoption rate of improved behaviors, children's reported diarrhea was reduced by 60 and 30% at 6 and 32 months post-intervention, respectively	(272)
Handwashing	Foodborne	Fingers –> food, fingers –> future victim	Handwashing at critical time points	Promotions of handwashing after defecation or after disposal of children's feces and before eating, preparing, or handling foods prevent around 25% of diarrhea episodes in LMIC settings	(273)
Improvement of water quality	Waterborne	Fluids –> food, fluids –> future victim	Source-based and point-of-use water improvements	Distributing disinfection products (chlorine products, flocculation and disinfection sachets) to households may reduce diarrhea by around 25% (RR 0.77, 95% CI 0.65–0.91 for chlorine products; RR 0.69, 95% CI 0.58–0.82 for flocculation and disinfection sachets). POU filtration systems may lower diarrhea episodes by around a half (RR: 0.48; 95% CI: 0.38–0.59). Proper application of solar disinfection may reduce diarrhea by around 30% (RR: 0.62; 95% CI: 0.42–0.94)	(274)

#### Animal Waste Management

Proper disposal and management of animal waste would not only reduce children's exposure to animal feces in the domestic environment but would also prevent the contamination of water sources and soil/fields by animal waste, both of which are proximal pathways depicted in the F-diagram. It might also reduce fly density in and around the household. Of the 29 records included, four articles examined the effect of animal waste management measures (biogas digesters and composting) on pathogen loads in animal manure, prior to being applied to fields (247–250). Two articles used pig manure, and one experimental study used poultry feces. One study did not specify the type of manure.

The increased temperature and biological activity during anaerobic digestion creates a hostile environment for enteric microorganisms, which contributes to the reduction of pathogen loads in the animal waste. Three studies investigated the performance of biogas digesters in small-scale households or farms in LMIC. Overall, biogas digesters of various design reduced the concentration of fecal indicator organisms (FIO) including enterococci, *E. coli*, and coliforms by 1–2.5 log_10_ CFU/mL.

One study (249) suggested that installation of a biogas digester was associated with a significant reduction in the count of FIO inside and outside of households in Ethiopia. However, the FIO counts were significantly increased on door handles of households with biogas digesters, indicating an increased risk of exposure of hands to fecal microorganisms by handling animal manure. Composting pig manure in clay-covered heaps is a typical practice of animal waste management in Vietnamese small-scale pig farms (250). Results showed that the concentration of *E. coli* in composted pig manure with 2% urea added was reduced from 4 log_10_ CFU/g to below the detection limit within 2 weeks. The counts of total coliforms decreased from 5.11 to 0.72 log_10_ CFU/g at day 45. However, *Enterococcus* spp. were not reduced by composting.

#### Corralling Small Animals

Three studies from the same study group evaluated the effectiveness of corralling chicken in households on mitigating infection and diarrhea associated with *C. jejuni* in children in a Peruvian peri-urban setting. A 2-month household trial testing the cultural and socioeconomic feasibility of corralling chickens in local communities (251) indicated that corralling didn't fully achieve the goal of separating children from poultry, as children in the household persistently played with (chickens in) the corrals, even with child-proof door latches. In addition, young children were responsible for animal care in certain households (251), which continually put them in contact with animals/feces. Besides perceptions about the connection between poultry and disease, additional food and water costs would be potential obstacles for a sustainable intervention in this setting (251). A randomized controlled trial suggested that corralling significantly increased (by 2-fold) the incidence of *Campylobacter*-related diarrhea in children living in the corral group compared to the control group (275). There was a non-significant decrease in asymptomatic infections in the corral group than in the control group [2.68 episodes per person per year (epy) vs. 3.12 epy]. Households were revisited 10 years after the intervention to evaluate their attitudes and poultry-raising practices. A significant drop in poultry-raising was observed, and 81% of households no longer kept chickens in their homesteads. However, the majority of the participants (92.3%) would prefer to use corrals in poultry-raising, referencing the cleanliness of the home as the most common reason (253).

A recent study piloted the use of animal hutches to provide a separate space for small animals in households in the Democratic Republic of the Congo (254). The hutches were constructed in kitchens and living quarters with three levels for different species of small animals. Rabbits and guinea pigs were placed in the top two levels separately day and night. The bottom level was for chickens and other poultry, which were only contained during the night. Participants reported that the construction of the animal hutches prevented rabbits and guinea pigs from defecating anywhere in the house, thus preventing children from eating animal feces on the floor. No studies on infection outcomes were performed.

#### Baby WaSH

Of the 29 studies, four reported tailored WaSH interventions for infants and young children to help reduce their exposure to feces from both humans and free-range livestock in rural smallholder families (BabyWaSH) (256). One possible intervention is to provide a safe and clean play area and feeding environment, such as a play-yard or playpen for children. Plastic manufactured play-yards, combined with caregiver education, handwashing, and safe water practices have been included in the WaSH intervention package used in the Sanitation, Hygiene, Infant Nutrition Efficacy (SHINE) trial in rural Zimbabwe. The package failed to reduce the prevalence and levels of enteric pathogens and to prevent EED in children (36, 255). A pilot study in rural Zambia (256) suggested a community-built play-yard vs. a plastic play-yard may be a feasible and acceptable alternative to plastic play-yards made in the USA (as used in SHINE) for local caregivers in LMIC, and the community-built play-yard protected the young children from ingesting soil and livestock feces. The formative research of the Reducing Enteropathy, Diarrhea, Undernutrition, and Contamination in the Environment (REDUCE) program evaluated the feasibility and acceptability of BabyWaSH interventions in the Democratic Republic of the Congo (254). Locally sourced playmats, tested as one Care Group Module prevented children from mouthing dirt.

#### Milk Hygiene

Milk and dairy products are critical, nutrient-dense components of a healthy diet, especially for children living in LMIC. However, the high nutrient concentrations also make them the ideal medium for the rapid growth of spoilage microorganisms and foodborne pathogens which is substantiated by storing products at ambient temperatures. Two review articles and two research articles reported on improving the microbiological safety of milk and dairy products.

Boiling or heating raw milk before consumption or further production of fermented dairy products is a workable practice to avoid the spoilage of milk (257, 258). Raw milk is heated up to around 65–80°C for about 30–50 min while producing fermented yogurt-like products in Ghana and Mali and can significantly reduce the levels pathogens in the raw milk. The application of pasteurization of raw milk (formally defined as 60–65°C for 30 m or 71–74°C for 15–40 s) can reduce pathogen levels including *E. coli, Salmonella, Listeria monocytogenes*, and *C. jejuni/coli* by a factor of 10^6^ (276).

Natural fermentation of raw milk is considered the cheapest and most convenient measure to extend the shelf-life of milk among African smallholder dairy farmers (257). Lactic acid bacteria (LAB) and yeasts involved in the fermentation process can considerably decrease the pH from approximately 6.5 to 3.0 after 24 h of fermentation. The reduced pH along with the release of antimicrobial compounds by fermenting bacteria synergistically kill or inhibit the growth of pathogenic microbes in the milk (277).

Pastoralists traditionally smoke the inner surface of plastic or wooden milk vessels by burning wood chips of specific trees and shrubs to disinfect after cleaning. A study in Kenya (259) found a significant difference of ~1 log_10_ cfu/cm^2^ in median microbial load between smoked containers and negative controls (only washed by plain water). Indicators included total viable count (TVC), total coliform count (TCC), and LAB. A qualitative study in Ethiopia also identified the smoking of milk-handling containers as one of potential risk mitigation practices in the pastoral communities (260). The researchers further assessed the effect of using stainless-steel containers on the microbial load of Ethiopian yogurt compared to the traditional smoking method with wooden containers and found no significant difference in microbial load of traditional yogurt between the two container types. Moreover, the pastoralists showed strong preference for the smoked traditional wooden containers over stainless-steel containers (275).

#### Complementary Food Hygiene

Twelve studies investigated food hygiene interventions related to proper preparation, handling, and feeding of complementary food for children. Of these 12 articles, two studies discussed protocols/practices of cleaning and disinfecting baby feeding bottles. An experimental study artificially contaminated baby bottles with fecal bacteria including enteropathogenic *Escherichia coli* (EPEC) and *Salmonella* spp. and then disinfected using different approaches (261). Rinsing baby bottles with soapy water followed by tap water reduced pathogen loads by 3.1–3.7 log_10_ cfu/mL of formula. Another study examined caregiver preferability of two bottle-cleaning methods using the improved practices (TIP) approach (262). Mothers and caregivers preferred brushing the bottle with dish detergent for 30 s after every use compared to boiling the bottle for several minutes daily.

The remaining 10 records included 3 cluster randomized controlled trials (cRCTs) (263, 264, 272), 1 cluster-randomized before-after study (265), 1 experimental study (270), and 5 observational studies (266–269, 271). These studies primarily evaluated the uptake of improved complementary food hygiene behaviors among mothers and caregivers in LMIC. Critical control points and motivational drivers of behavior change were determined based on theoretical frameworks. These intervention programs usually developed measures targeting multiple food hygiene behaviors including: (1) handwashing before cooking; (2) handwashing before feeding children; (3) washing cooking utensils with safe water and soap and drying them on a clean and elevated surface; (4) proper and safe storage of cooked food and utensils; (5) thorough reheating food before feeding; (6) boiling children's drinking water. Seven of these studies only measured the adoption of recommended behaviors, and the results suggested a varying degree of increase in targeted complementary food hygiene behaviors. Two articles (270, 271) evaluated the effects of improved hygiene practices on reduction of microorganisms in foods and found a significant reduction in fecal contamination of the food samples 3 weeks after training of mothers. Only one RCT measured not only the intermediate outcome (adoption rate of improved behavior) but also a health-related outcome (272); children's diarrhea was significantly reduced by 60 and 30% at 6 months and 32 months post-intervention, respectively, suggesting noteworthy short and long-term effects of the community-level intervention.

#### Handwashing

Handwashing has been identified as one of the critical target behaviors that help interrupt fecal-oral transmission. One recent systematic review assessed the effects of handwashing promotion on preventing diarrhea in children and adults based on the results of 29 randomized controlled trials published before January 2020, of which 15 RCTs are community-based trials in LMIC with 29,347 participants (273). The handwashing promotion interventions consisted of various health education activities, along with provision of free soap in some trials. The major time points for handwashing targeted in the trials included after defecation or after disposal of children's feces and before eating, preparing, or handling foods. Handwashing prevented ~25% of diarrhea episodes.

#### Improvement of Water Quality

One systematic review summarized studies evaluating interventions to improve the microbial quality of drinking water to prevent diarrhea (274). A total of 55 studies published before November 2014 were included in this systematic review; 50 were conducted in LMIC. The study types included cRCTs, quasi-RCTs, and controlled before-and-after studies. The primary outcome of interest in most trials was self-reported diarrhea. Interventions to improve the water quality were divided into two categories: source-based improvements and point-of-use (POU) interventions. In LMIC settings, the source-based interventions included providing protected ground water, communal tap stands, and improved community water supplies through chlorination or filtration. POU interventions consisted of chlorination, flocculation, installation of filtration systems, and solar disinfection. Current evidence was not sufficient to determine whether source-based interventions reliably reduced diarrhea, whereas POU water treatment measures demonstrated varying effects on reducing diarrhea episodes. Distributing disinfection products to households may reduce diarrhea by around 25%. POU filtration systems, specifically ceramic filters, biosand systems and LifeStraw filters, may lower diarrhea episodes by around a half. Proper application of SODIS (having filled bottles exposed to direct sunlight for at least 6 h before drinking) may reduce diarrhea by around 30%. No studies have evaluated the impact of POU water treatment on asymptomatic infections.

## Discussion

### Benefits of Smallholder Livestock Production

To understand the risks and benefits of ASF from livestock production, we started with a robust understanding of the literature on the benefits of livestock production. We reviewed the three primary pathways through which livestock production could benefit child nutritional outcomes: production, income, and empowerment.

#### Production

This review found overall positive impacts of livestock production interventions on child and household dietary outcomes. Because improved livestock production can generate income and increase the availability of nutrient-rich ASF, livestock production interventions can have important implications for smallholder households compared to education-only or other non-production-focused interventions that aim to increase ASF consumption (73). Furthermore, the focus for livestock production interventions should not be on production or education alone, as increased production does not always lead to increased utilization or consumption of ASF (57, 116, 278–280); integrating an evidence-based educational or BCC strategy as part of the intervention may facilitate better nutritional and health outcomes, particularly for women and children (122). Benefits from livestock production interventions can occur rapidly but have the potential to strengthen over time, so coupling livestock production interventions with education, technical assistance, and/or BCC may also have implications for long-term sustainability. This may be particularly relevant to women and children, as the dynamics of intrahousehold ASF allocation can be complex (281). Translating production into optimal consumption of ASF requires a combination of efforts, including promoting optimal livestock keeping practices to maintain a healthy herd size that can meet both consumption and income needs and addressing sociocultural and gendered norms and practices (83).

#### Income

The contribution of livestock production to household income may be limited for low-income households as a result of constraints in their access to markets, input and output services, hired labor, and high productivity animals (86). Evidence from three reviews points to the complexity of the allocation of income from livestock production, suggesting that livestock play multiple intersecting roles in smallholder farming households (116, 280, 282). There is some evidence that households engaged in livestock production may have to meet a certain income or livestock production threshold prior to livestock production significantly benefitting the household's nutrition (86, 98, 109, 283, 284).

Furthermore, the availability of and access to markets may cause a shift toward sale of livestock for income generation, as smallholder farmers are integrated into the cash economy and face competitive prices for livestock products (57, 285). Markets, however, also provide access to complementary resources that contribute to overall health and well-being, such education, health services, and other non-food items (89, 286). The presence of markets may also contribute to the sustainability and scalability of livestock production interventions, as livestock production may have positive implications for the local food environment when ASF are produced and sold locally; however, the results are mixed and suggest stronger support for livestock ownership at the household level and improved ASF consumption (73, 81). More research is needed to understand how to optimize dietary intake through integration into local markets.

Separating the effects of the livestock production and income pathways on dietary intake can be challenging, as the income pathway may be dependent on the production pathway (70). Their distinction, however, may be important for households that use livestock production primarily for their own consumption, as production for own-consumption can occur alongside production for sale as households adapt to changing agricultural and market conditions (287).

#### Empowerment

There is some evidence that livestock production interventions with women as the primary recipients have beneficial effects on women's empowerment. Livestock represent a store of value and wealth for rural smallholder households, and women tend to be more engaged in poultry production than in other livestock production activities (282, 288). Women are crucial actors in food systems, generally playing a major role in caring for and managing livestock, and are often the primary decision-makers about child diet and healthcare (288). Thus, addressing women's empowerment in livestock interventions has important potential for improving their ability to care for their children through facilitated earning potential, control over resources, and autonomy over decisions (75, 77, 288, 289). Increased women's empowerment, such as increasing control over production or ensuring ownership of livestock, may also have important implications for child nutrition (127, 290).

Evidence suggests that interventions incorporating BCC strategies into livestock production interventions may be more effective at changing aspects of women's empowerment than those that do not. The benefits of livestock production may not accrue to the most vulnerable within the household (which are often women and children). Furthermore, supporting livestock production by women is not an automatic route to empowerment, so livestock production interventions coupled with evidence-based BCC strategies have the potential to improve children's health outcomes through improving human and social capital, enhancing women's knowledge about production, improving production outcomes, reducing risk of diseases, and facilitating sustainability of intervention impacts over time (21, 77, 122, 290).

However, the use of different measures of dietary outcomes and women's empowerment have limited the comparability of interventions (75). Moreover, it has been argued that the use of quantitative women's empowerment measures in development research, such as the WEAI, often excludes local meanings, values, and features of empowerment, producing results that may not reflect local conceptualizations of empowerment (291). As such, livestock production interventions that aim to empower women may not be structured in relevant or sustainable ways for communities (292). Rigorous evaluations of intervention design and consistent measurements of outcomes coupled with impact pathway analyses are needed to continue to build a robust evidence base on how livestock production interventions can be designed and implemented to best contribute to women's empowerment, gender equity, and sustained improved child health and nutrition outcomes (126).

### Risks of Smallholder Livestock Production

#### Enteric Pathogen Infections, Impaired Gut Health, and Undernutrition

Findings from the MAL-ED study suggested consumption of foods with insufficient energy and proteins, combined with *asymptomatic* enteric pathogen infection were key drivers of undernutrition. In seeking to understand stunting, researchers have suggested that EED, as an outcome of asymptomatic gut infection, may be the “missing piece” (47, 49). Following this body of thought, we explicitly incorporate impaired gut health into the long-established UNICEF framework as earlier recommended (47). Conversely, both acute (wasting) and chronic (stunting) undernutrition are risk factors for diarrhea and other infectious diseases, amplifying the “vicious cycle of diseases of poverty” (293). We found that many enteric pathogens have been associated with EED and/or undernutrition outcomes. These epidemiological associations are supported by evidence from experimental studies in laboratory animals, suggesting that the combination of enteric pathogen infections and deficient diets may lead to EED and undernutrition (188). The relative risks of enteric pathogen infection differ between species or genera. Many species or genera of enteric pathogens, alone or in combination, have been associated with increased EED markers and/or growth impairment. At the population level, risks are mainly associated with those pathogens that occur at high prevalence in infants and young children. These include zoonotic pathogens, notably *Campylobacter* species and to a lesser extent *Giardia* and *Cryptosporidium* species. Among *Campylobacter* species, the impact of poorly studied non-thermotolerant (“emerging”) species such as the *C. hyointestinalis* and *C. fetus* groups have been associated with higher risks than the established thermotolerant species *C. jejuni* and *C. coli*. Thus, the full benefits of improved nutrition through livestock production can only be realized by concurrently averting CU5 infection with zoonotic enteric pathogens.

#### Risks of Exposure to Zoonotic Enteric Pathogens and Their Attribution

Utilizing studies that have evaluated the association between animal ownership and (a)symptomatic health outcomes without measuring the pathogens themselves (294–296), we reviewed risk factors in/around the smallholder household and their association with zoonotic pathogens in the household/farm environment, livestock, or humans. Results indicate risk of exposure through the animal contact pathway, but also point to direct/indirect exposure of CU5 from other pathways related to WaSH, infant and young child feeding, general household hygiene, or food safety. Our review found that exposure assessments embedded in these studies and the identification of putative exposure sources were based on assumptions regarding dominant exposure routes that were not quantified. Future epidemiological or interventional studies should incorporate microbiological attribution technologies to strengthen the measurement or control of risks.

### Risk Mitigation

There are numerous mitigation strategies to prevent transmission of zoonotic pathogens from livestock to infants and young children, yet very few of them have been tested in LMIC and even fewer are based on quantified contribution of different reservoirs and pathways to transmission. Among the possible options, many have additional benefits, including animal health, reusing waste products, and energy generation.

Reducing the transmission of zoonotic pathogens at the source by minimizing the dissemination of animal feces to broader environments is a powerful option. Biogas digesters are a low-cost and promising measure in LMIC settings, as they can effectively reduce the pathogen loads in animal waste while simultaneously generating energy for cooking and lighting. Biogas may replace the use of dung patties as cooking fuel, a practice that negatively affects indoor air quality and women and children's health (249, 297–299). Other methods of processing animal manure for safety, e.g., before applying as a fertilizer, have not yet been studied in the context of LMIC.

Measures to improve the microbial safety and quality of milk and milk products, such as boiling, natural fermentation, and smoking milk-handling containers, should continue to be encouraged in the local communities. Milking hygiene and udder health also play important roles, and interventions on milking hygiene to control mastitis should be emphasized and further studied to help reduce pathogens in the milk (300). More broadly, improved animal health management may help reduce the exposure of livestock to animal and zoonotic pathogens. For example, providing a hygienic and comfortable environment for livestock and reducing pathogen levels in feed bunks and communal water supplies can interrupt the horizontal and vertical transmission of pathogens among livestock (301, 302).

Although corralling chicken failed to reduce the *Campylobacter*-associated diarrhea in children in one study site, this type of intervention should not be dismissed, as contact between children and chickens was not fully controlled in these studies. Furthermore, dynamic models have suggested that when taking acquired immunity to *Campylobacter* into consideration, a moderate decrease in the force of infection may lead to an increase in diarrhea incidence (303, 304). As illustrated in this review, current evidence indicates that most *Campylobacter* infections are asymptomatic without overt diarrhea but cannot be neglected given the considerable long-term impacts on children. We suggest that the effectiveness of interventions in preventing asymptomatic colonization of enteric pathogens should also be included in the design of future interventions among children under five in LMIC.

The results of behavior-focused studies suggest that significant improvements in both individual and community level food hygiene behaviors can be achieved through theory-driven BCC approaches. However, few interventions have targeted intermediate endpoints (e.g., microbial loads) or child health outcomes; current studies are more formative in nature. Additional research is needed to evaluate the downstream effects of behavior change, to ensure that changes in caregivers' behaviors translate to improvements in child health.

### Strengths and Limitations

Animal ownership presents competing risks and benefits to smallholder households, through animal feces exposure and nutrition supply (46). By including authors from the fields of social science, microbiology, epidemiology, and library science, we were able to review a broad array of literature to synthesize the empirical risk-benefit evidence on this topic. Though data synthesis as seen in meta-analyses was not feasible given the diversity of study designs, our novel approach had team members collectively synthesize narrative results, putting into dialogue findings across disciplines to understand the full scope of benefits and risks of SLP on human nutrition. To our knowledge, this is the first system-level risk-benefit analysis of SLP and child nutrition.

Our study had several limitations. The etiology of EED and stunting is highly complex, and focusing on specific risk factors, as in this review, may lead to oversimplification. Also, the association between enteric pathogens and undernutrition may be mediated through other disease pathways, such as anemia, whose association with livestock husbandry has been investigated in a previous review (305). Study designs and population characteristics of included studies were highly heterogeneous, limiting our ability to infer the strength of evidence of the associations reviewed in these studies and to draw direct comparisons across some results and intervention designs. Some empirical evidence included in the review comes from a reduced modeling approach, which may limit the effect of endogenous factors on outcomes such as decision-making, thereby biasing results and complicating interpretation. We also omitted non-English full texts in this review due to language capacity of the authors and time constraints, making the results vulnerable to publication bias.

In this review, we aimed to conduct a risk-benefit analysis. We acknowledge that our system-level analysis, from smallholder livestock production to child nutrition, is a sub-set of relations within a broader system, as depicted within the UNICEF framework's basic, underlying conditions. While the interdisciplinary approach to include benefits and risks expands the boundaries of dynamics examined, this, like any effort to understand a bounded part of a system, may be biased and/or inconsistent.

The level of pathogen speciation in many of the epidemiological studies in section Risks factors of exposure to or infection with zoonotic enteric pathogens associated with smallholder livestock production was limited, as they defined endpoints of human infection by pathogen groups rather than at the genus, species, or subtype level. This complicates inference about putative reservoirs of these pathogens as anthroponotic, zoonotic, or sapronotic. Further source attribution studies, particularly for pathogens with both anthroponotic and zoonotic reservoirs, are recommended. Furthermore, such studies should aim to quantify the relative importance of sources and transmission pathways so that interventions can be appropriately directed. The SaniPath analytical approach, one of the few examples of quantification, relied on *E. coli* as an indicator of fecal contamination, but could not differentiate sources at the reservoir level. We concur with the authors who suggested addressing this limitation by combining the tool with other attribution approaches (244). There is also a need to adapt the tool, originally developed for urban settings with a focus on human waste, to rural settings focusing on animal waste.

Very few studies address specific interventions to reduce exposure to zoonotic pathogens in LMIC, particularly in smallholder environments. Lacking quantitative assessment of the relative weights of pathogen reservoirs and pathways, available studies on control measures focus on selected transmission pathways without evidence that the targeted pathways are the main contributors to exposure. Future intervention studies should be guided by source attribution estimates and quantitative exposure assessment to select the most promising sites for intervention.

## Conclusion

**Figure 2:**
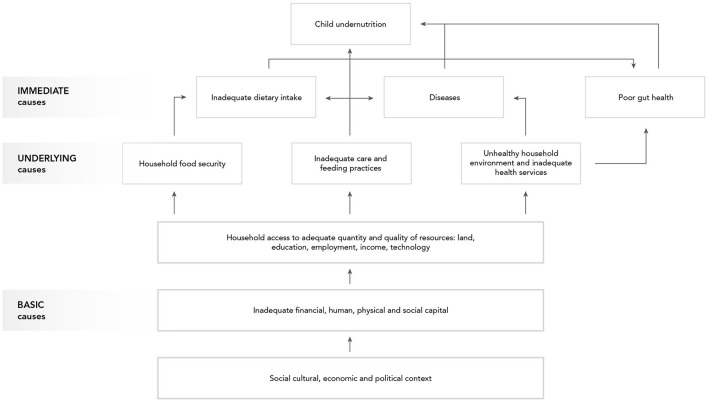
Modified framework for child undernutrition.

There has recently been a call for a One Health approach to strengthen efforts to improve child nutrition by taking into account enteric pathogens from animal feces, which remains a neglected element in mainstream WaSH interventions (306). The literature in this review extends this call to fully evaluate the benefits and risks of livestock production on child nutrition and supports the inclusion of poor gut health, manifested as EED, into the UNICEF framework of child undernutrition (see Figure 2). The attribution of zoonotic enteric infection in children to certain sources and implementation of corresponding control measures could forestall the risks, resulting in symptomatic diseases and poor gut health, and maximize the benefits, conduce to adequate diet intake, brought by the SLP.

## Author Contributions

SM, AH, DC, XL, and KM conceptualized the topic. With significant methodological support from NS, DC, XL, and KM specified keywords and search strategies, and conducted literature search, screenings, and data extraction. DC, XL, and KM synthesized the evidence and drafted portions of the manuscript, with substantial conceptual guidance from AH and SM. All authors contributed to the drafting of the final manuscript.

## Funding

This work was funded by the United States Agency for International Development (USAID) Bureau for Food Security under Agreement #AID-OAA-L-15-00003 as part of Feed the Future Innovation Lab for Livestock Systems, and by the Bill & Melinda Gates Foundation OPP#1175487.

The use of Covidence for this publication was supported by the University of Florida Clinical and Translational Science Institute, which was supported in part by the NIH National Center for Advancing Translational Sciences under award number UL1TR001427.

## Author Disclaimer

Any opinions, findings, conclusions, or recommendations expressed here are those of the authors alone. The content is solely the responsibility of the authors and does not necessarily represent the official views of the funders.

## Conflict of Interest

The authors declare that the research was conducted in the absence of any commercial or financial relationships that could be construed as a potential conflict of interest.

## Publisher's Note

All claims expressed in this article are solely those of the authors and do not necessarily represent those of their affiliated organizations, or those of the publisher, the editors and the reviewers. Any product that may be evaluated in this article, or claim that may be made by its manufacturer, is not guaranteed or endorsed by the publisher.
