# Supplementary material for: Benefits and Risks of Smallholder Livestock Production on Child Nutrition in Low- and Middle-Income Countries
Source: Front Nutr. 2021 Oct 27;8:751686. doi: 10.3389/fnut.2021.751686 (PMC8579112; doi:10.3389/fnut.2021.751686)
Supplement: Supplementary file 1 [file Table_1.docx]

***Supplementary Tables of Benefits and Risks of Smallholder Livestock Production on Child Nutrition in Low- and Middle- Income Countries (LMIC)***

**S 1 Table. P-Values and/or 95% Confidence Intervals of Primary or Relevant Outcome Variables Measured in Livestock Production Interventions**

|  | | | Primary or Relevant Outcome Variables Measured | | | | | |
| --- | --- | --- | --- | --- | --- | --- | --- | --- |
|  |  |  | P-value and/or (95% CI) | | | | |  |
| Reference | Study country | Intervention/Educational Component | Production | ASF consumption | Dietary intake | Child nutritional status | Income & expenditure | Women’s Empowerment |
| (1) | Ethiopia | African Chicken Genetic Gains (ACGC)/BCC | Egg production: ACGC* vs control: P < 0.01; ATONU* vs control: P < 0.05 | Child egg consumption: ATONU vs control: P < 0.01; ACGC/ATONU vs ACGC: P < 0.05 | Child DD*: ACGC/ATONU vs ACGC: P < 0.05 | HAZ, WAZ, WHZ*: NS between intervention groups | Chicken income: ACGC vs control: P < 0.01; ATONU vs. control: P < 0.01 | Women’s decision making related to chickens: ATONU vs control: P < 0.10 |
| (2) | Nigeria | African Chicken Genetic Gains (ACGC)/BCC | P < 0.05 | HH* ASF consumption: P < 0.05 | N/A | N/A | P < 0.05 | N/A |
| (3) | Burkina Faso | *Un Oeuf*/BCC | Poultry production: P < 0.05 | Child ASF consumption: P < 0.05 | N/A | Wasting: P = 0.03, underweight: P = 0.02 | N/A | Women’s decision-making about eggs: P < 0.05 |
| (4) | Nicaragua | Production Support for Agri-Food Program (APAGRO)/Technical assistance | N/A | N/A | HH food availability & HH food access: P < 0.1; HH food stability: P < 0.05 | N/A | Income from livestock sales: reported significant difference, no P-value reported | WEAI*: P < 0.05 |
| (5) | Zambia | Realigning Agriculture for Improving Nutrition (RAIN)/BCC | P < 0.01 | HH dairy consumption: P < 0.01 | NS | N/A | N/A | N/A |
| (6) | Ethiopia | Gifting eggs/ BCC | Significance not reported | Child egg consumption: P < 0.001 | N/A | N/A | N/A | N/A |
| (7) | Zambia | Realigning Agriculture for Improving Nutrition (RAIN)/BCC | N/A | N/A | N/A | Mean WHZ: P < 0.05 | N/A | Social capital, asset access, financial empowerment, agricultural empowerment: P < 0.05 |
| (8) | Bolivia | Egg production | N/A | Child egg consumption: P < 0.0001 | N/A | N/A | N/A | N/A |
| (9) | Nepal | Enhanced-Homestead Food Production (E-HFP)/BCC | Egg production: NS | N/A | % HH food insecure: (51.0-56.1) | NS | N/A | N/A |
| (10) | Zambia | Community Markets for Conservation (COMACO) | Poultry production: NS | HH ASF consumption: NS | N/A | N/A | Significance not reported | N/A |
| (11) | Zambia | Copperbelt Rural Livelihoods Enhancement Support Project (CRLESP) | N/A | N/A | Probability-weighted DDS*: P < 0.01 | N/A | Income/expenditure from livestock: P < 0.01 | N/A |
| (12) | Burkina Faso | HKI Enhanced-Homestead Food Production (E-HFP)/BCC | N/A | N/A | HH DD: NS | N/A | N/A | Overall empowerment score: P < 0.01 |
| (13) | Burkina Faso | HKI E-HFP/BCC | N/A | N/A | HH DDS: P = 0.07; child minimum DD: P = 0.08 | Wasting: P = 0.08 | N/A | N/A |
| (14) | India | Integrated Child Development Services (ICDS)/BCC | N/A | HH egg consumption: P < 0.001 | N/A | Moderate-to-severe malnutrition: P < 0.01 Ljung Box test | N/A | N/A |
| (15) | Rwanda | Heifer International Livestock Donation | N/A | HH dairy consumption: P < 0.01; HH meat consumption: P < 0.10 | Respondent DD: P < 0.01 | WAZ: P < 0.10; WHZ: P < 0.05 | N/A | N/A |
| (16) | Bangladesh | Strengthening the Dairy Value Chain Project (SDVCP) | N/A | N/A | N/A | N/A | N/A | Variable impact on aspects of women’s empowerment |
| (17) | Cambodia | HKI HFP | Poultry production: significance NR | HH ASF consumption: significance not reported | N/A | N/A | Income from sale of poultry: reported significant difference, no P-value reported | N/A |
| (18) | Bangladesh, Cambodia, Philippines, Nepal | HKI HFP/BCC | N/A | Child egg consumption: P < 0.05; HH egg consumption: P < 0.05 | N/A | N/A | HH income from sale of livestock production: P < 0.05 | N/A |
| (19) | Bangladesh | HKI HFP/BCC | Food varieties grown: significant NR | HH egg consumption: significance not reported | N/A | N/A | N/A | Income decisionmakers: significance NR |
| (20) | Bangladesh | HFP/BCC | N/A | N/A | HH DDS: significance not reported | N/A | N/A | N/A |
| (21) | Kenya | National Agriculture and Livestock Extension Program (NALEP) | Mean milk production: P < 0.00001 | Child milk consumption: P < 0.001; HH milk consumption: P < 0.001 | N/A | N/A | Monthly HH income: P < 0.05 | N/A |
| (22) | Cambodia | HKI HFP/BCC | N/A | Child egg consumption: P < 0.05 | HH DDS: P < 0.05 | NS | NS | N/A |
| (23) | Myanmar |  | N/A | HH consumption of chicken meat: NS | N/A | N/A | Income from sale of animals: P < 0.001 | N/A |
| (24) | Ethiopia | Dairy Goat Development Project (DGDP) | Increased egg production: significance not reported | Child ASF consumption: significance not reported | N/A | N/A | Sale of eggs: significance not reported | N/A |
| (25) | Ethiopia | Dairy Goat Development Project (DGDP) | N/A | HH ASF consumption: significance not reported | N/A | WHZ, WAZ, HAZ: significance NR | N/A | N/A |
| (26) | Bangladesh | Participatory Livestock Development Project (PLDP) | Egg production: P < 0.001 | Child ASF consumption: NS | Child intake of food groups: NS | N/A | Number of eggs sold: P < 0.001 | N/A |

N/A denotes that the outcome was not measured in the study. NS denotes that the outcome was measured and reported as not significant.

*ACGC, African Chicken Genetics Gains. ATONU, Agriculture to Nutrition. DD, dietary diversity. HAZ, height-for-age z-score. WAZ, weight-for-age z-score. WHZ, weight for height z-score. HH, household. WEAI, women’s empowerment in agriculture index. DDS, dietary diversity score.

**S 2 Table. Enteric pathogens associated with increased risk of Environmental Enteric Dysfunction (EED) at the 95% confidence level and their effect sizes**

| Pathogen group | Genus/common name | Species | Subtype | Reservoir^*^(Ref.) | Affected gut function^&^ | Marker of gut function^&^ | Effect size (unit) | P-value and/or  (95% CI) | Ref. |
| --- | --- | --- | --- | --- | --- | --- | --- | --- | --- |
| Viruses | Adenovirus |  |  | A (27) | Gut inflammation | MPO | (0, 0.25)^#^ | <0.05 | (28) |
|  | Norovirus |  |  | A (29) | N/A | N/A | N/A | N/A | N/A |
|  | Rotavirus |  |  | A (30) | N/A | N/A | N/A | N/A | N/A |
| Bacteria | *Aeromonas* | spp. |  | S (31) | Epithelial damage/repair | AAT | (-0.5, -0.25)^#^ | <0.05 | (28) |
|  | *Campylobacter* | spp. |  | Z (32) | Epithelial damage/repair, gut inflammation | MPO,  AAT,  NEO, | MPO: +0.18 (ng/mL), NEO: −0.12 (nmol/L),  AAT: +0.05 (mg/g) | MPO: (0.13, 0.22),  AAT: (0.02, 0.09),  NEO: (−0.16, −0.08) | (33) |
|  |  |  |  |  | Gut inflammation, epithelial damage/repair, intestinal permeability | MPO, NEO, AAT, L:M | MPO: (0, 0.25)^#^,  NEO: (-0.25, 0)^#^, AAT: (0, 0.25)^#^, L:M: (0, 0.25)^#^ | <0.05 | (28) |
|  |  |  |  |  | Gut inflammation | MPO | +7751.2 (ng/mL) | 0.03 | (34) |
|  |  | *jejuni/coli* |  | Z (32) | N/A | N/A | N/A | N/A | N/A |
|  | *Escherichia* | *coli* | Enterotoxigenic | A (35) | Gut inflammation | CAL | +1.35 $(\mu g/g)$ | (1.005, 1.82) | (36) |
|  |  |  |  |  | Gut inflammation | MPO | +7089.2 (ng/mL) | 0.019 | (34) |
|  |  |  |  |  | Gut inflammation | MPO, NEO | MPO: (0, 0.25) ^#^, NEO: (-0.25, 0)^#^ | <0.05 | (28) |
|  |  |  | Enteroaggregative | A (35) | Gut inflammation | Lactoferrin | +1.8 (fold-rise) | 0.019 | (37) |
|  |  |  |  |  | Gut inflammation | MPO | +0.14 [ln(ng/mL)] | (0.11, 0.18) | (38) |
|  |  |  |  |  | Gut inflammation | MPO | 0.33 ($\beta$ coefficient) | 0.02 | (39) |
|  |  |  |  |  | Gut inflammation, epithelial damage/repair | MPO, NEO, AAT | MPO: (0, 0.25) ^#^, NEO: (0, 0.25) ^#^, AAT: (0, 0.25)^#^ | <0.05 | (28) |
|  |  |  | Enteroinvasive | A (35) | Gut inflammation, intestinal permeability | MPO, L:M | MPO: (0, 0.25) ^#^, L:M: (0.25, 0.5)^#^ | <0.05 | (28) |
|  |  |  | Enteropathogenic | A (35) | Epithelial damage/repair | AAT | (-0.25, 0)^#^ | <0.05 | (28) |
|  | *Helicobacter* | *pylori* |  | A (40) | Epithelial damage/repair | AAT,  Reg1$\beta$ | AAT: 0.26 ($\beta$ coefficient),  Reg 1$\beta$: -0.32 ($\beta$ coefficient) | AAT: 0.03 (0.02, 0.49),  Reg 1$\beta$: 0.02 (-0.59, -0.05) | (41) |
|  | *Plesiomonas* | *shigelloides* |  | S (42) | Gut inflammation | MPO | (0.25, 0.5)^#^ | <0.05 | (28) |
|  | *Salmonella* | spp. |  | A/Z (43) | Epithelial damage/repair | AAT | (-0.5, -0.25)^#^ | <0.05 | (28) |
|  | *Shigella* | spp. |  | A (44) | Gut inflammation, intestinal permeability | MPO, L:M | MPO: (0.25, 0.5) ^#^, L:M: (0.25, 0.5)^#^ | <0.05 | (28) |
|  | *Yersinia* | *enterocolitica* |  | Z (45) | Gut inflammation | MPO, NEO | MPO: (0.25, 0.5) ^#^, NEO: (-0.5, -0.25)^#^ | <0.05 | (28) |
| Protozoa | *Cryptosporidium* | spp. |  | A/Z (46) | Epithelial damage/repair | I-FABP | 1.35 ($\beta$ coefficient) | <0.001 | (47) |
|  |  |  |  |  | Intestinal permeability | L:M | (0.25, 0.5) ^#^ | <0.05 | (28) |
|  |  | *hominis* |  | A (46) | N/A | N/A | N/A | N/A | N/A |
|  |  | *parvum* |  | A/Z (48) | N/A | N/A | N/A | N/A | N/A |
|  | *Entamoeba* | *histolytica* |  | A (49) | N/A | N/A | N/A | N/A | N/A |
|  | *Giardia* | spp. |  | A/Z (50) | Gut inflammation, epithelial damage/repair | MPO, AAT, NEO | MPO: 0.55 ($\beta$ coefficient), AAT: 0.34 ($\beta$ coefficient),  EE score: 1.51 ($\beta$ coefficient) | MPO: 0.008 (0.15, 0.95)  , AAT: 0.03 (0.04, 0.63)  EE score: <0.001 (0.79–2.23) | (39) |
|  |  |  |  |  | Epithelial damage/repair, microbial translocation | Reg 1$\beta$,  Flic IgA | Reg 1$\beta$: +72.189 $(\mu g/g)$,  Flic IgA: +0.054 (optical density) | Reg 1$\beta$: 0.006,  Flic IgA: 0.0091 | (34) |
|  |  |  |  |  | Gut inflammation, intestinal permeability | MPO, NEO, L:M | MPO: (-0.25, 0) ^#^, NEO: (-0.25, 0)^#^, L:M: (0, 0.25)^#^ | <0.05 | (28) |
|  |  |  |  |  | Intestinal permeability | L:M | 0.22 (difference in L:M Z-score) | (0.12, 0.32) | (51) |
|  |  | *lamblia* |  | A/Z (50) | Intestinal permeability | L:M | Infected: 0·43 (ratio) vs. non-infected: 0·25 (ratio) | 0.014 | (52) |
|  |  |  |  |  |  |  |  |  |  |
| geo helminths |  |  |  | A^^^ | N/A | N/A | N/A | N/A | N/A |
|  | *Ascaris* | *lumbricoides* |  | A (53) | N/A | N/A | N/A | N/A | N/A |
|  | Hookworm |  |  | A (54) | N/A | N/A | N/A | N/A | N/A |
|  | *Trichuris* | spp. |  | A (53) | Gut inflammation, epithelial damage/repair | MPO, AAT, NEO | NEO: 0.90 ($\beta$ coefficient),  EE score: 1.71 ($\beta$ coefficient) | NEO: 0.01 (0.19, 1.61),  EE score: 0.02 (0.32, 3.11) | (39) |
|  |  | *trichiura* |  | A (53) | N/A | N/A | N/A | N/A | N/A |

*A: anthroponotic; Z: zoonotic; A/Z: anthroponotic/zoonotic.

^&^Markers or scheme in original literature measuring the gut functions: myeloperoxidase (MPO), neopterin (NEO), calprotectin (CAL), lactoferrin – gut inflammation; regenerating protein 1$\beta$ (Reg 1$\beta$), $\alpha$-1-antitrypsin (AAT), intestinal fatty acid binding protein (I-FABP) – epithelial damage/repair; lactulose:mannitol (L:M) – intestinal permeability; anti-flic immunoglobulin A (Flic IgA) – microbial translocation; EE score: a composite score of MPO, NEO, and AAT – gut inflammation and epithelial damage/repair.

#The reviewed article applied a heatmap to summarize the effect of pathogens on markers studied. Since the study depicted effect by color gradient that was broken into four intervals – (-0.5, -0.25), (-0.25, 0), (0, 0.25), and (0.25, 0.5), we visually evaluated and reported the interval that an effect size belonged instead.

^^^In the reviewed literature, geohelminths consisted of *Ascaris*, *Trichuris*, hookworm.

**S 3 Table. Enteric pathogens associated with increased risk of undernutrition at the 95% confidence level and their effect sizes**

| Pathogen group | Genus/common name | Species | Subtype | Reservoir^*^(Ref.) | Undernutrition indicator^&^ | Effect size (unit) | P-value and/or  (95% CI) | Ref. |
| --- | --- | --- | --- | --- | --- | --- | --- | --- |
| Viruses | Adenovirus |  |  | A (27) | HAZ | Symptomatic: -0.36 (Z-score) | Symptomatic: < 0.001 | (55) |
|  | Norovirus |  |  | A (29) | HAZ | Symptomatic: -0.10 (Z-score) | Symptomatic: < 0.001 | (55) |
|  | Rotavirus |  |  | A (30) | HAZ | Symptomatic: -0.26 (Z-score) | Symptomatic: < 0.001 | (55) |
| Bacteria | *Aeromonas* | spp. |  | S (31) | N/A | N/A | N/A | N/A |
|  | *Campylobacter* | spp. |  | Z (32) | weight | Symptomatic: -43.9 (g)^&^; asymptomatic -65.5 (g)^&^ | Symptomatic: 0.049  (-87.6, -1.0);  asymptomatic 0.040 (-128.0, -3.0) | (56) |
|  |  |  |  |  | LAZ | -1.82 (Z-score) at high burden vs. −1.49 (Z-score) at low burden | (-1.94, -1.70) at high burden vs.  (-1.60, -1.38) at low burden | (33) |
|  |  |  |  |  | LAZ | Asymptomatic: -0.17 (Z-score) | Asymptomatic: (-0.32, -0.01) | (57) |
|  |  | *jejuni/coli* |  | Z (32) | LAZ | -0.11 (SD)^&^ | 0.003 | (58) |
|  |  |  |  |  | LAZ | Symptomatic: −0.16 (Z-score) | Symptomatic:  (-0.32, -0.01) | (59)^#^ |
|  | *Escherichia* | *coli* | Enterotoxigenic | A (35) | Malnutrition (WAZ<-2) | 2.7 (RR)^&^, 0.40 (AR), 63 (AP among malnourished) | (1.1– 6.9) (RR), (0.03, 0.70) (AR) | (60) |
|  |  |  |  |  | LAZ | -0.15 (SD)^&^ | <0.001 | (58) |
|  |  |  | Enteroaggregative | A (35) | LAZ | -0.30 (Z-score) | (-0.44, -0.16) | (38) |
|  |  |  |  |  | LAZ | Asymptomatic: -0.21 (Z-score) | Asymptomatic: (–0.37, –0.05) | (61) |
|  |  |  | Enteroinvasive | A (35) | N/A | N/A | N/A | N/A |
|  |  |  | Enteropathogenic | A (35) | N/A | N/A | N/A | N/A |
|  | *Helicobacter* | *pylori* |  | A (40) | N/A | N/A | N/A | N/A |
|  | *Plesiomonas* | *shigelloides* |  | S (42) | N/A | N/A | N/A | N/A |
|  | *Salmonella* | spp. |  | A/Z (43) | N/A | N/A | N/A | N/A |
|  | *Shigella* | spp. |  | A (44) | height | Symptomatic: 0.055 cm less growth/percent days | Symptomatic: 0.008 | (62) |
|  |  |  |  |  | LAZ | Asymptomatic: -0.14 (Z-score) | Asymptomatic: (–0.27, –0.01) | (61) |
|  | *Yersinia* | *enterocolitica* |  | Z (45) | N/A | N/A | N/A | N/A |
| Protozoa | *Cryptosporidium* | spp. |  | A/Z (46) | Malnutrition (WAZ<-2) | 1.7 (RR)^&^, 0.20 (AR), 40 (AP among malnourished) | (1.1, 2.6) (RR), (0.03, 0.38) (AR) | (60) |
|  |  |  |  |  | LAZ | -0.26 in India (Z-score), -0.20 in Bangladesh | India: (-0.51, -0.01), Bangladesh: (-0.44, -0.05) | (63) |
|  |  |  |  |  | WAVZ, LAVZ | -WAVZ: -0.43 (Z-score), -LAVZ: -0.55 (Z-score) | -WAVZ: 0.023, -LAVZ:0.005 | (64) |
|  |  | *hominis*^$^ |  | A (46) | Severe stunting | 2.69  (OR) | 0.019 (1.17, 6.15) | (65)^$^ |
|  |  |  |  |  | Stunting, underweight, wasting | Stunting: 1.65 (OR), underweight: 2.08 (OR), wasting: 2.04 (OR) | Stunting: (1.06, 2.57), underweight: (1.34, 3.22), wasting: (1.21, 3.43) | (66)^$^ |
|  |  | *parvum* |  | A/Z (48) | Stunting, underweight, wasting | Stunting: 1.22 (OR), underweight: 1.30 (OR), wasting: 1.24 (OR) | Stunting: (1.04, 1.44), underweight: (1.11, 1.54), wasting: (1.05, 1.46) | (67) |
|  | *Entamoeba* | *histolytica* |  | A (49) | WAZ | -0.37 (Z-score) | 0.03 | (68) |
|  |  |  |  |  | Malnutrition (WAZ<-2) | 1.89 (RR)^&^, 0.25 (AR), 47 (AP among malnourished) | (1.2, 3.0) (RR), (0.06, 0.44) (AR) | (60) |
|  | *Giardia* | spp. |  | A/Z (50) | LAZ | -0.17 (Z-score) | (–0.30, -0·05) | (61) |
|  |  |  |  |  | WAZ | -WAZ: -0.29 (Z-score) | -WAZ: (−0.53, −0.05) | (51) |
|  |  |  |  |  | LAZ | -0.4 (Z-score) | 0.05 (−0.80, −0.001) | (69) |
|  |  | *lamblia* |  | A/Z (50) | Stunting, | 1.6 (OR) | 0 (1.2, 2.1) | (70) |
|  |  |  |  |  | WAZ,  WHZ, wasting | -WAZ: -0.69 (Z-score), -WHZ: -0.41 (Z-score),  wasting: 5.42 (OR) | -WAZ: 0.002,  -WHZ: 0.003, wasting: <0.001 (2.97, 9.89) | (71) |
|  |  |  |  |  | Stunting | 1.67 (OR) | (1.12, 2.49) | (72) |
|  |  |  |  |  | LAZ | -0.10 (Z-score) | 0.047 (-0.21, -0.00) | (73) |
|  |  |  |  |  | Undernutrition (MUAC<12.5 cm) | 3.50 (OR) | (2.21, 5.54) | (74) |
|  |  |  |  |  | LAZ,  LAD | -LAZ: -0.10 (Z-score),  -LAD: -0.32 | -LAZ: 0.018,  -LAD: 0.013 | (64) |
| Geohelminths |  |  |  | A^^^ | LAZ,  LAD | -LAZ: -0.16  (Z-score),  -LAD: -0.48 | -LAZ: <0.001 (-0.25, -0.07),  -LAD: <0.001 (-0.76, -0.20) | (64) |
|  |  |  |  |  | Stunting | -0.84 ($\beta$ coefficient) | (−1.48, −0.20) | (75) |
|  | *Ascaris* | *lumbricoides* |  | A (53) | HAZ | -0.60 ($\beta$ coefficient) | 0.007 (−1.03, −0.16) | (71) |
|  | Hookworm |  |  | A (54) | LAZ | -0.36 (Z-score) | 0.001 | (68) |
|  | *Trichuris* | spp. |  | A (53) | N/A | N/A | N/A | N/A |
|  |  | *trichiura* |  | A (53) | Stunting | 2.4 (OR) | (1.1, 5.3) | (76) |

*A: anthroponotic; Z: zoonotic; A/Z: anthroponotic/zoonotic.

^&^Full names of the abbreviations: RR – risk ratio, SD: standard deviation, g – gram, AR – attributed risk, AP – attributed proportion, cm – centimeter, LAD – length-for-age difference, LAZ: length-for-age Z-score, LAVZ: length velocity Z-score, WAZ: weight-for-age Z-score, WAVZ: weight velocity Z-score.

^$^*Cryptosporidium hominis* as the dominant species of *C*. spp in the affected populations.

^^^In the reviewed literature, geohelminths consisted of *Ascaris*, *Trichuris*, hookworm.

**References**

1. Passarelli S, Ambikapathi R, Gunaratna NS, Madzorera I, Canavan CR, Noor AR, Worku A, Berhane Y, Abdelmenan S, Sibanda S, et al. A Chicken Production Intervention and Additional Nutrition Behavior Change Component Increased Child Growth in Ethiopia: A Cluster-Randomized Trial. *J Nutr* (2020) **150**:2806–2817. doi:10.1093/jn/nxaa181

2. Alabi OO, Ajayi FO, Bamidele O, Yakubu A, Ogundu EU, Sonaiya EB, Ojo MA, Hassan WA, Adebambo OA. Impact assessment of improved Chicken genetics on livelihoods and food security of smallholder poultry farmers in Nigeria. *Livest Res Rural Dev* (2020) **32**:

3. McKune SL, Stark H, Sapp AC, Yang Y, Slanzi CM, Moore E V, Omer A, Wereme N’Diaye A. Behavior Change, Egg Consumption, and Child Nutrition: A Cluster Randomized Controlled Trial. *Pediatrics* (2020) **146**: doi:10.1542/peds.2020-007930

4. Salazar L, Fahsbender J, Kim N. Livestock transfers, food security and women’s empowerment: evidence from a randomized phased-in program in Nicaragua. *IDB Work Pap Ser - Inter-American Dev Bank* (2018)

5. Rosenberg AM, Maluccio JA, Harris J, Mwanamwenge M, Nguyen PH, Tembo G, Rawat R. Nutrition-sensitive agricultural interventions, agricultural diversity, food access and child dietary diversity: Evidence from rural Zambia. *Food Policy* (2018) **80**:10–23. doi:https://doi.org/10.1016/j.foodpol.2018.07.008

6. Omer A, Mulualem D, Classen H, Vatanparast H, Whiting SJ. A community poultry intervention to promote egg and eggshell powder consumption by young children in Halaba Special Woreda, SNNPR, Ethiopia. *J Agric Sci* (2018) **10**:

7. Kumar N, Nguyen PH, Harris J, Harvey D, Rawat R, Ruel MT. What it takes: evidence from a nutrition- and gender-sensitive agriculture intervention in rural Zambia. *J Dev Eff* (2018) **10**:341–372. doi:10.1080/19439342.2018.1478874

8. Berti PR, Araujo Cossio H. Raising chickens for increased egg consumption in a rural highland Bolivian population. *Food Secur* (2017) **9**:1329–1341. doi:10.1007/s12571-017-0728-5

9. Osei A, Pandey P, Nielsen J, Pries A, Spiro D, Davis D, Quinn V, Haselow N. Combining Home Garden, Poultry, and Nutrition Education Program Targeted to Families With Young Children Improved Anemia Among Children and Anemia and Underweight Among Nonpregnant Women in Nepal. *Food Nutr Bull* (2016) **38**:49–64. doi:10.1177/0379572116676427

10. Dumas S, Lungu L, Mulambya N, Daka W, McDonald E, Steubing E, Lewis T, Backel K, Jange J, Lucio-Martinez B, et al. Sustainable smallholder poultry interventions to promote food security and social, agricultural, and ecological resilience in the Luangwa Valley, Zambia. *Food Secur* (2016) **8**:507–520. doi:10.1007/s12571-016-0579-5

11. Jodlowski M, Winter-Nelson A, Baylis K, Goldsmith PD. Milk in the data: food security impacts from a livestock field experiment in Zambia. *World Dev* (2016) **77**:99–114. doi:10.1016/j.worlddev.2015.08.009

12. Olney DK, Bliznashka L, Pedehombga A, Dillon A, Ruel MT, Heckert J. A 2-Year Integrated Agriculture and Nutrition Program Targeted to Mothers of Young Children in Burkina Faso Reduces Underweight among Mothers and Increases Their Empowerment: A Cluster-Randomized Controlled Trial. *J Nutr* (2016) **146**:1109–1117. doi:10.3945/jn.115.224261

13. Olney DK, Pedehombga A, Ruel MT, Dillon A. A 2-Year Integrated Agriculture and Nutrition and Health Behavior Change Communication Program Targeted to Women in Burkina Faso Reduces Anemia, Wasting, and Diarrhea in Children 3–12.9 Months of Age at Baseline: A Cluster-Randomized Controlled Trial. *J Nutr* (2015) **145**:1317–1324. doi:10.3945/jn.114.203539

14. Murty PVVS, Rao MV, Bamji MS. Impact of Enriching the Diet of Women and Children Through Health and Nutrition Education, Introduction of Homestead Gardens and Backyard Poultry in Rural India. *Agric Res* (2016) **5**:210–217. doi:10.1007/s40003-016-0206-x

15. Rawlins R, Pimkina S, Barrett CB, Pedersen S, Wydick B. Got milk? The impact of Heifer International’s livestock donation programs in Rwanda on nutritional outcomes. *Food Policy* (2014) **44**:202–213. doi:https://doi.org/10.1016/j.foodpol.2013.12.003

16. Quisumbing AR, Roy S, Njuki J, Kakuly T, Waithanji E. Can dairy value-chain projects change gender norms in rural Bangladesh? Impacts on assets, gender norms, and time use. *IFPRI - Discuss Pap* (2013)

17. Olney DK, Vicheka S, Kro M, Chakriya C, Kroeun H, Hoing LS, Talukder A, Quinn V, Iannotti L, Becker E, et al. Using Program Impact Pathways to Understand and Improve Program Delivery, Utilization, and Potential for Impact of Helen Keller International’s Homestead Food Production Program in Cambodia. *Food Nutr Bull* (2013) **34**:169–184. doi:10.1177/156482651303400206

18. Talukder A, Haselow NJ, Osei AK, Villate E, Reario D, Kroeun H, SokHoing L, Uddin A, Dhunge S, Quinn V. Homestead food production model contributes to improved household food security and nutrition status of young children and women in poor populations: Lessons learned from scaling-up programs in Asia (Bangladesh, Cambodia, Nepal and Philippines). *J F Actions* (2010)

19. Iannotti L, Cunningham K, Ruel M. “Diversifying into healthy diets: homestead food production in Bangladesh.,” in *Millions fed: proven successes in agricultural development* (Washington: International Food Policy Research Institute), 145–151.

20. Langworthy M, Caldwell R. Save the Children USA in Bangladesh: Jibon O Jibika Program: Endline Survey Report. (2009).

21. Walingo MK. Role of livestock projects in empowering women smallholder farmers for sustainable food security in rural Kenya. *African J Food, Agric Nutr Dev* (2009) **9**:1468–1483.

22. Olney DK, Talukder A, Iannotti LL, Ruel MT, Quinn V. Assessing Impact and Impact Pathways of a Homestead Food Production Program on Household and Child Nutrition in Cambodia. *Food Nutr Bull* (2009) **30**:355–369. doi:10.1177/156482650903000407

23. Henning J, Morton J, Pym R, Hla T, Meers J. Evaluation of strategies to improve village chicken production: controlled field trials to assess effects of Newcastle disease vaccination and altered chick rearing in Myanmar. *Prev Vet Med* (2009) **90**:17–30. doi:10.1016/j.prevetmed.2009.04.007

24. Ayele Z, Peacock C. Improving Access to and Consumption of Animal Source Foods in Rural Households: The Experiences of a Women-Focused Goat Development Program in the Highlands of Ethiopia. *J Nutr* (2003) **133**:3981S-3986S. doi:10.1093/jn/133.11.3981S

25. Habtemariam K, Ayalew W, Habte GZ, Meske G. Enhancing the role of livestock production in improving nutritional status of farming families: Lessons from a dairy goat development project in Eastern Ethiopa. *Livest Res Rural Dev* (2003) **15**:

26. Nielsen H, Roos N, Thilsted SH. The Impact of Semi-Scavenging Poultry Production on the Consumption of Animal Source Foods by Women and Girls in Bangladesh. *J Nutr* (2003) **133**:4027S-4030S. doi:10.1093/jn/133.11.4027S

27. Lynch JP, Kajon AE. Adenovirus: Epidemiology, Global Spread of Novel Serotypes, and Advances in Treatment and Prevention. *Semin Respir Crit Care Med* (2016) **37**:586–602. doi:10.1055/S-0036-1584923

28. Kosek MN, Ahmed T, Bhutta ZA, Caulfield L, Guerrant RL, Houpt E, Kang G, Lee G, Lima AAM, McCormick BJJ, et al. Causal Pathways from Enteropathogens to Environmental Enteropathy: Findings from the MAL-ED Birth Cohort Study. *EBioMedicine* (2017) doi:10.1016/j.ebiom.2017.02.024

29. Koopmans M. Progress in understanding norovirus epidemiology. *Curr Opin Infect Dis* (2008) **21**:544–552. doi:10.1097/QCO.0B013E3283108965

30. Haffejee IE. The epidemiology of rotavirus infections: a global perspective. *J Pediatr Gastroenterol Nutr* (1995) **20**:275–286.

31. Fernández-Bravo A, Figueras MJ. An Update on the Genus Aeromonas: Taxonomy, Epidemiology, and Pathogenicity. *Microorganisms* (2020) **8**: doi:10.3390/MICROORGANISMS8010129

32. Kaakoush NO, Castaño-Rodríguez N, Mitchell HM, Man SM. Global epidemiology of campylobacter infection. *Clin Microbiol Rev* (2015) **28**:687–720. doi:10.1128/CMR.00006-15

33. Amour C, Gratz J, Mduma ER, Svensen E, Rogawski ET, McGrath M, Seidman JC, McCormick BJJ, Shrestha S, Samie A, et al. Epidemiology and Impact of Campylobacter Infection in Children in 8 Low-Resource Settings: Results from the MAL-ED Study. *Clin Infect Dis* (2016) doi:10.1093/cid/ciw542

34. Iqbal NT, Syed S, Kabir F, Jamil Z, Akhund T, Qureshi S, Liu J, Ma JZ, Guleria S, Gewirtz A, et al. Pathobiome driven gut inflammation in pakistani children with environmental enteric dysfunction. *PLoS One* (2019) doi:10.1371/journal.pone.0221095

35. Wasteson Y. Zoonotic Escherichia coli. *Acta Vet Scand Suppl* (2001) **95**:79–84. Available at: https://pubmed.ncbi.nlm.nih.gov/11995395/ [Accessed June 9, 2021]

36. George CM, Burrowes V, Perin J, Oldja L, Biswas S, Sack D, Ahmed S, Haque R, Bhuiyan NA, Parvin T, et al. Enteric Infections in Young Children are Associated with Environmental Enteropathy and Impaired Growth. *Trop Med Int Heal* (2018) doi:10.1111/tmi.13002

37. Opintan JA, Newman MJ, Ayeh-Kumi PF, Affrim R, Gepi-Attee R, Sevilleja JEAD, Roche JK, Nataro JP, Warren CA, Guerrant RL. Pediatric diarrhea in Southern Ghana: Etiology and association with intestinal inflammation and malnutrition. *Am J Trop Med Hyg* (2010) doi:10.4269/ajtmh.2010.09-0792

38. Rogawski ET, Guerrant RL, Havt A, Lima IFN, Medeiros PHQS, Seidman JC, McCormick BJJ, Babji S, Hariraju D, Bodhidatta L, et al. Epidemiology of enteroaggregative Escherichia coli infections and associated outcomes in the MAL-ED birth cohort. *PLoS Negl Trop Dis* (2017) doi:10.1371/journal.pntd.0005798

39. Fahim SM, Das S, Gazi MA, Mahfuz M, Ahmed T. Association of intestinal pathogens with faecal markers of environmental enteric dysfunction among slum-dwelling children in the first 2 years of life in Bangladesh. *Trop Med Int Heal* (2018) doi:10.1111/tmi.13141

40. LM B. Helicobacter pylori: epidemiology and routes of transmission. *Epidemiol Rev* (2000) **22**:283–297. doi:10.1093/OXFORDJOURNALS.EPIREV.A018040

41. Fahim SM, Das S, Gazi MA, Alam MA, Hasan MM, Hossain MS, Mahfuz M, Masudur Rahman M, Haque R, Sarker SA, et al. Helicobacter pylori infection is associated with fecal biomarkers of environmental enteric dysfunction but not with the nutritional status of children living in Bangladesh. *PLoS Negl Trop Dis* (2020) doi:10.1371/journal.pntd.0008243

42. Michael Janda J, Abbott SL, McIver CJ. Plesiomonas shigelloides revisited. *Clin Microbiol Rev* (2016) **29**:349–374. doi:10.1128/CMR.00103-15

43. Giannella RA. Salmonella. *Med Microbiol* (1996) Available at: https://www.ncbi.nlm.nih.gov/books/NBK8435/ [Accessed July 6, 2021]

44. Faherty CS, Lampel KA. “Shigella,” in *Food Microbiology: Fundamentals and Frontiers* (wiley), 317–345. doi:10.1128/9781555819972.ch12

45. Bottone EJ. Yersinia enterocolitica: Overview and epidemiologic correlates. *Microbes Infect* (1999) **1**:323–333. doi:10.1016/S1286-4579(99)80028-8

46. Leitch GJ, He Q. Cryptosporidiosis-an overview. *J Biomed Res* (2011) **25**:1–16. doi:10.1016/S1674-8301(11)60001-8

47. Amadi B, Zyambo K, Chandwe K, Besa E, Mulenga C, Mwakamui S, Siyumbwa S, Croft S, Banda R, Chipunza M, et al. Adaptation of the small intestine to microbial enteropathogens in Zambian children with stunting. *Nat Microbiol* (2021) doi:10.1038/s41564-020-00849-w

48. Dillingham RA, Lima AA, Guerrant RL. Cryptosporidiosis: epidemiology and impact. *Microbes Infect* (2002) **4**:1059–1066. doi:10.1016/S1286-4579(02)01630-1

49. Ackers JP, Mirelman D. Progress in research on Entamoeba histolytica pathogenesis. *Curr Opin Microbiol* (2006) **9**:367–373. doi:10.1016/j.mib.2006.06.014

50. Fantinatti M, Bello AR, Fernandes O, Da-Cruz AM. Identification of Giardia lamblia Assemblage e in Humans Points to a New Anthropozoonotic Cycle. *J Infect Dis* (2016) **214**:1256–1259. doi:10.1093/infdis/jiw361

51. Rogawski ET, Bartelt LA, Platts-Mills JA, Seidman JC, Samie A, Havt A, Babji S, Trigoso DR, Qureshi S, Shakoor S, et al. Determinants and impact of Giardia infection in the first 2 years of life in the MAL-ED birth cohort. *J Pediatric Infect Dis Soc* (2017) doi:10.1093/jpids/piw082

52. Goto R, Panter-Brick C, Northrop-Clewes CA, Manahdhar R, Tuladhar NR. Poor intestinal permeability in mildly stunted Nepali children: associations with weaning practices and Giardia lamblia infection. *Br J Nutr* (2002) doi:10.1079/bjn2002599

53. Anthroponoses. in *Encyclopedia of Parasitology* (Springer Berlin Heidelberg), 89–89. doi:10.1007/978-3-540-48996-2_211

54. Hotez PJ, Brooker S, Bethony JM, Bottazzi ME, Loukas A, Xiao S. Hookworm Infection. *N Engl J Med* (2004) **351**:799–807. doi:10.1056/NEJMra032492

55. Bray AE, Ahmed S, Das SK, Khan SH, Chisti MJ, Ahmed T, Faruque ASG, Fuchs GJ. Viral pathogen-specific clinical and demographic characteristics of children with moderate-to-severe diarrhea in Rural Bangladesh. *Am J Trop Med Hyg* (2019) doi:10.4269/ajtmh.19-0152

56. Lee G, Pan W, Peñataro Yori P, Paredes Olortegui M, Tilley D, Gregory M, Oberhelman R, Burga R, Chavez CB, Kosek M. Symptomatic and Asymptomatic Campylobacter Infections Associated with Reduced Growth in Peruvian Children. *PLoS Negl Trop Dis* (2013) doi:10.1371/journal.pntd.0002036

57. Rogawski ET, Liu J, Platts-Mills JA, Kabir F, Lertsethtakarn P, Siguas M, Khan SS, Praharaj I, Murei A, Nshama R, et al. Use of quantitative molecular diagnostic methods to investigate the effect of enteropathogen infections on linear growth in children in low-resource settings: longitudinal analysis of results from the MAL-ED cohort study. *Lancet Glob Heal* (2018) **6**:E1319–E1328. doi:10.1016/S2214-109x(18)30351-6

58. Platts-Mills JA, Gratz J, Mduma E, Svensen E, Amour C, Liu J, Maro A, Saidi Q, Swai N, Kumburu H, et al. Association between stool enteropathogen quantity and disease in Tanzanian children using TaqMan Array Cards: A nested case-control study. *Am J Trop Med Hyg* (2014) doi:10.4269/ajtmh.13-0439

59. Schnee AE, Haque R, Taniuchi M, Uddin MJ, Alam MM, Liu J, Rogawski ET, Kirkpatrick B, Houpt ER, Petri WA, et al. Identification of Etiology-Specific Diarrhea Associated with Linear Growth Faltering in Bangladeshi Infants. *Am J Epidemiol* (2018) doi:10.1093/aje/kwy106

60. Mondal D, Haque R, Sack RB, Kirkpatrick BD, Petri WA Jr. Attribution of malnutrition to cause-specific diarrheal illness: evidence from a prospective study of preschool children. *Am J Trop Med Hyg* (2009)

61. Rogawski ET, Liu J, Platts-Mills JA, Kabir F, Lertsethtakarn P, Siguas M, Khan SS, Praharaj I, Murei A, Nshama R, et al. Use of quantitative molecular diagnostic methods to investigate the effect of enteropathogen infections on linear growth in children in low-resource settings: longitudinal analysis of results from the MAL-ED cohort study. *Lancet Glob Heal* (2018) doi:10.1016/S2214-109X(18)30351-6

62. Lee G, Olortegui MP, Yori PP, Black RE, Caulfield L, Chavez CB, Hall E, Pan WK, Meza R, Kosek M. Effects of shigella-, campylobacter- and ETEC-associated diarrhea on childhood growth. *Pediatr Infect Dis J* (2014) doi:10.1097/INF.0000000000000351

63. Korpe PS, Valencia C, Haque R, Mahfuz M, McGrath M, Houpt E, Kosek M, McCormick BJJ, Penataro Yori P, Babji S, et al. Epidemiology and Risk Factors for Cryptosporidiosis in Children from 8 Low-income Sites: Results from the MAL-ED Study. *Clin Infect Dis* (2018) **67**:1660–1669. doi:10.1093/cid/ciy355

64. Garzón M, Pereira-da-Silva L, Seixas J, Papoila AL, Alves M. Subclinical enteric parasitic infections and growth faltering in infants in São Tomé, Africa: A birth cohort study. *Int J Environ Res Public Health* (2018) doi:10.3390/ijerph15040688

65. Korpe PS, Haque R, Gilchrist C, Valencia C, Niu F, Lu M, Ma JZ, Petri SE, Reichman D, Kabir M, et al. Natural History of Cryptosporidiosis in a Longitudinal Study of Slum-Dwelling Bangladeshi Children: Association with Severe Malnutrition. *PLoS Negl Trop Dis* (2016) doi:10.1371/journal.pntd.0004564

66. Delahoy MJ, Omore R, Ayers TL, Schilling KA, Blackstock AJ, Ochieng JB, Moke F, Jaron P, Awuor A, Okonji C, et al. Clinical, environmental, and behavioral characteristics associated with Cryptosporidium infection among children with moderate-to-severe diarrhea in rural western Kenya, 2008–2012: The Global Enteric Multicenter Study (GEMS). *PLoS Negl Trop Dis* (2018) doi:10.1371/journal.pntd.0006640

67. Tumwine JK, Kekitiinwa A, Nabukeera N, Akiyoshi DE, Rich SM, Widmer G, Feng X, Tzipori S. Cryptosporidium parvum in children with diarrhea in Mulago Hospital, Kampala, Uganda. *Am J Trop Med Hyg* (2003) doi:10.4269/ajtmh.2003.68.710

68. LaBeaud AD, Nayakwadi Singer M, McKibben M, Mungai P, Muchiri EM, McKibben E, Gildengorin G, Sutherland LJ, King CH, King CL, et al. Parasitism in Children Aged Three Years and Under: Relationship between Infection and Growth in Rural Coastal Kenya. *PLoS Negl Trop Dis* (2015) doi:10.1371/journal.pntd.0003721

69. Donowitz JR, Alam M, Kabir M, Ma JZ, Nazib F, Platts-Mills JA, Bartelt LA, Haque R, Petri WA. A Prospective Longitudinal Cohort to Investigate the Effects of Early Life Giardiasis on Growth and All Cause Diarrhea. *Clin Infect Dis* (2016) doi:10.1093/cid/ciw391

70. J.H. B-G, G.M. G-M, D. G-P, D.C. A-A, M.C. A-U. Giardia intestinalis and nutritional status in children participating in the complementary nutrition program, Antioquia, Colombia, May to October 2006. *Rev Inst Med Trop Sao Paulo* (2009)

71. Aiemjoy K, Gebresillasie S, Stoller NE, Shiferaw A, Tadesse Z, Chanyalew M, Aragie S, Callahan K, Keenan JD. Epidemiology of soil-transmitted helminth and intestinal protozoan infections in preschool-aged children in the Amhara Region of Ethiopia. *Am J Trop Med Hyg* (2017) doi:10.4269/ajtmh.16-0800

72. Caron Y, Hong R, Gauthier L, Laillou A, Wieringa FT, Berger J, Poirot E. Stunting, beyond acute diarrhoea: Giardia duodenalis, in Cambodia. *Nutrients* (2018) **10**: doi:10.3390/nu10101420

73. Lehto KM, Fan YM, Oikarinen S, Nurminen N, Hallamaa L, Juuti R, Mangani C, Maleta K, Hyöty H, Ashorn P. Presence of Giardia lamblia in stools of six- to 18-month old asymptomatic Malawians is associated with children’s growth failure. *Acta Paediatr Int J Paediatr* (2019) doi:10.1111/apa.14832

74. Osman KA, Zinsstag J, Tschopp R, Schelling E, Hattendorf J, Umer A, Ali S, Cercamondi CI. Nutritional status and intestinal parasites among young children from pastoralist communities of the Ethiopian Somali region. *Matern Child Nutr* (2020) doi:10.1111/mcn.12955

75. Gyorkos TW, Maheu-Giroux M, Casapía M, Joseph SA, Creed-Kanashiro H. Stunting and helminth infection in early preschool-age children in a resource-poor community in the Amazon lowlands of Peru. *Trans R Soc Trop Med Hyg* (2011) doi:10.1016/j.trstmh.2010.12.003

76. Rabaoarisoa CR, Rakotoarison R, Rakotonirainy NH, Mangahasimbola RT, Randrianarisoa AB, Jambou R, Vigan-Womas I, Piola P, Randremanana RV. The importance of public health, poverty reduction programs and women’s empowerment in the reduction of child stunting in rural areas of Moramanga and Morondava, Madagascar. *PLoS One* (2017) doi:10.1371/journal.pone.0186493
