# Supplementary material for: Benefits and Risks of Smallholder Livestock Production on Child Nutrition in Low- and Middle-Income Countries
Source: Front Nutr. 2021 Oct 27;8:751686. doi: 10.3389/fnut.2021.751686 (PMC8579112; doi:10.3389/fnut.2021.751686)
Supplement: Supplementary file 2 [file Data_Sheet_1.docx]

***Supplementary Appendix of Benefits and Risks of Smallholder Livestock Production on Child Nutrition in Low- and Middle- Income Countries (LMIC)***

Table of Contents

[1 Supplementary information 1 2](#_Toc78534856)

[1.1 Sample (PubMed) search strategies for questions studying benefits of smallholder livestock production* 2](#_Toc78534857)

[1.1.1 Search strategy for question 1 2](#_Toc78534858)

[1.1.2 Search strategy for question 2 3](#_Toc78534859)

[1.1.3 Search strategy for question 3 3](#_Toc78534860)

[1.2 Sample (PubMed) search strategies for questions studying risks of smallholder livestock production* 4](#_Toc78534861)

[1.2.1 Search strategy for question 1 4](#_Toc78534862)

[1.2.2 Search strategy for question 2 5](#_Toc78534863)

[1.2.3 Search strategy for question 3 7](#_Toc78534864)

[2 Supplementary information 2 13](#_Toc78534865)

[2.1 Eligibility criteria for literature studying the benefits of smallholder livestock production 13](#_Toc78534866)

[2.1.1 Eligibility criteria for literature studying question 1 13](#_Toc78534867)

[2.1.2 Eligibility criteria for literature studying question 2 14](#_Toc78534868)

[2.1.3 Eligibility criteria for literature studying question 3 14](#_Toc78534869)

[2.2 Eligibility criteria for literature studying the risks of smallholder livestock production 14](#_Toc78534870)

[2.2.1 Eligibility criteria for literature studying question 1 14](#_Toc78534871)

[2.2.2 Eligibility criteria for literature studying question 2 15](#_Toc78534872)

[2.2.3 Eligibility criteria for literature studying question 3 16](#_Toc78534873)

[3 Supplementary information 3 18](#_Toc78534874)

[3.1 Quantitative summary of search results and screening process 18](#_Toc78534875)

*Search strategies for additional databases available from corresponding author upon request

# Supplementary information 1

## Sample (PubMed) search strategies for questions studying benefits of smallholder livestock production

### Search strategy for question 1

(("Livestock"[Mesh] OR "Poultry"[Mesh] OR "Sheep, Domestic"[Mesh] OR "Buffaloes"[Mesh] OR "Reindeer"[Mesh] OR "Goats"[Mesh] OR "Columbiformes"[Mesh] OR "Anseriformes"[Mesh] OR "Galliformes"[Mesh] OR "Chickens"[Mesh] OR "Turkeys"[Mesh] OR "Camelidae"[Mesh] OR "Rabbits"[Mesh] OR "Horses"[Mesh] OR "Guinea Pigs"[Mesh] OR "farm animal*"[tiab] OR "domestic animal*"[tiab] OR "domesticated animal*"[tiab] OR livestock[tiab] OR horse*[tiab] OR donkey*[tiab] OR cattle[tiab] OR cow[tiab] OR cows[tiab] OR ox[tiab] OR oxen[tiab] OR zebu*[tiab] OR bali*[tiab] OR yak[tiab] OR yaks[tiab] OR "water buffalo*"[tiab] OR gayal*[tiab] OR sheep*[tiab] OR goat*[tiab] OR reindeer*[tiab] OR pig[tiab] OR pigs[tiab] OR hog[tiab] OR hogs[tiab] OR swine[tiab] OR chicken*[tiab] OR duck*[tiab] OR poultr*[tiab] OR geese[tiab] OR goose[tiab] OR buffalo*[tiab] OR "domestic fowl*"[tiab] OR fowl*[tiab] OR camel*[tiab] OR llama*[tiab] OR alpaca*[tiab] OR rabbit*[tiab] OR "guinea pig*"[tiab] OR turkey*[tiab] OR pigeon*[tiab] OR porcine[tiab] OR bovine*[tiab] OR mule*[tiab] OR foal*[tiab] OR calf[tiab] OR calves[tiab] OR gosling*[tiab] OR duckling*[tiab] OR piglet*[tiab]) **AND** ("Animal Husbandry"[Mesh] OR smallhold*[tiab] OR "small hold*"[tiab] OR "small-hold*"[tiab] OR "small-scale farm*"[tiab] OR "small scale farm*"[tiab] OR "small-scale agriculture*"[tiab] OR "small scale agriculture*"[tiab] OR subsistence[tiab] OR "subsistence farm*"[tiab] OR "subsistence farming"[tiab] OR "family farm"[tiab] OR "pastoral"[tiab] OR "herding"[tiab] OR "agro-pastoral"[tiab] OR "agropastoral"[tiab] OR "agro pastoral"[tiab])) **AND** (("Feeding Behavior"[Mesh] OR "Food Preferences"[Mesh] OR "Diet, Healthy"[Mesh] OR "Breast Feeding"[Mesh] OR "diversified diet*" [tiab] OR "varied diet*" [tiab] OR "infant and young child feeding practice*"[tiab] OR "dietary diversity"[tiab] OR "dietary intake"[tiab] OR "complementary feeding"[tiab] OR "breast feed*"[tiab] OR breastfeed*[tiab] OR "breast fed"[tiab] OR "diet pattern*"[tiab] OR "dietary pattern*"[tiab] OR "complementary feeding"[tiab] OR "feeding behavior"[tiab]) **AND** ("Meat"[Mesh] OR "Meat Products"[Mesh] OR "Red Meat"[Mesh] OR "Pork Meat"[Mesh] OR "Meat Proteins"[Mesh] OR "Poultry"[Mesh] OR "Poultry Products"[Mesh] OR "Poultry Proteins"[Mesh] OR "Dietary Proteins"[Mesh] OR "Animal Proteins, Dietary"[Mesh] OR "Egg Proteins, Dietary"[Mesh] OR "Milk"[Mesh] OR "Milk Proteins"[Mesh] OR "animal source food*"[tiab] OR "ASF"[tiab] OR "animal source food consumption"[tiab] OR "ASF consumption"[tiab] OR milk[tiab] OR egg[tiab] OR eggs[tiab] OR meat*[tiab] OR beef[tiab] OR mutton[tiab] OR lamb[tiab] OR pork[tiab] OR protein[tiab] OR "vitamin A"[tiab] OR "vitamin B12"[tiab] OR "essential fatty acid*"[tiab] OR macronutrient*[tiab] OR micronutrient*[tiab] OR "dietary supplement*"[tiab] OR "infant nutrition"[tiab] OR "child nutrition"[tiab] OR "infant and child nutrition"[tiab] OR "animal food product*"[tiab] OR nutrition[tiab] OR offal[tiab] OR poultry[tiab] OR "poultry product*"[tiab] OR "poultry protein*"[tiab] OR "meat product*"[tiab] OR "meat protein*"[tiab] OR "pork meat*"[tiab] OR "red meat*"[tiab] OR "dietary protein*"[tiab] OR "milk protein*"[tiab])) AND (2000:2021[pdat]) AND (english[Filter])

Additional databases searched: **CABI** and EbscosHost’s **Academic Search Premier** and **CINAHL** (Cumulative Index of Nursing and Allied Health Information).

### Search strategy for question 2

(("Livestock"[Mesh] OR "Poultry"[Mesh] OR "Sheep, Domestic"[Mesh] OR "Buffaloes"[Mesh] OR "Reindeer"[Mesh] OR "Goats"[Mesh] OR "Columbiformes"[Mesh] OR "Anseriformes"[Mesh] OR "Galliformes"[Mesh] OR "Chickens"[Mesh] OR "Turkeys"[Mesh] OR "Camelidae"[Mesh] OR "Rabbits"[Mesh] OR "Horses"[Mesh] OR "Guinea Pigs"[Mesh]) OR "farm animal*"[tiab] OR "domestic animal*"[tiab] OR "domesticated animal*"[tiab] OR livestock[tiab] OR horse*[tiab] OR donkey*[tiab] OR cattle[tiab] OR cow[tiab] OR cows[tiab] OR ox[tiab] OR oxen[tiab] OR zebu*[tiab] OR bali*[tiab] OR yak[tiab] OR yaks[tiab] OR "water buffalo*"[tiab] OR gayal*[tiab] OR sheep*[tiab] OR goat*[tiab] OR reindeer*[tiab] OR pig[tiab] OR pigs[tiab] OR hog[tiab] OR hogs[tiab] OR swine[tiab] OR chicken*[tiab] OR duck*[tiab] OR poultr*[tiab] OR geese[tiab] OR goose[tiab] OR buffalo*[tiab] OR "domestic fowl*"[tiab] OR fowl*[tiab] OR camel*[tiab] OR llama*[tiab] OR alpaca*[tiab] OR rabbit*[tiab] OR "guinea pig*"[tiab] OR turkey*[tiab] OR pigeon*[tiab] OR porcine[tiab] OR bovine*[tiab] OR mule*[tiab] OR foal*[tiab] OR calf[tiab] OR calves[tiab] OR gosling*[tiab] OR duckling*[tiab] OR piglet*[tiab]) **AND** ("Animal Husbandry"[Mesh OR smallhold*[tiab] OR "small hold*"[tiab] OR "small-hold*"[tiab] OR "small-scale farm*"[tiab] OR "small scale farm*"[tiab] OR "small-scale agriculture*"[tiab] OR "small scale agriculture*"[tiab] OR subsistence[tiab] OR "subsistence farm*"[tiab] OR "subsistence farming"[tiab] OR "family farm"[tiab] OR "pastoral"[tiab] OR "herding"[tiab] OR "agro-pastoral"[tiab] OR "agropastoral"[tiab] OR "agro pastoral"[tiab])) **AND** (income [tiab] OR "income generation" [tiab] OR "household income" [tiab] OR "economic status" [tiab] OR poverty [tiab] OR wealth [tiab] OR asset* [tiab] OR livelihood* [tiab] OR "household livelihood*" [tiab] OR "livelihood generation" [tiab]) **AND** ("infant and young child feeding practice*"[tiab] OR diet[tiab] OR "dietary diversity"[tiab] OR "dietary intake*"[tiab] OR "diet intake"[tiab] OR "diet pattern*"[tiab] OR "dietary pattern*"[tiab] OR "complementary feeding"[tiab] OR "supplementation"[tiab] OR "supplement*"[tiab] OR "infant and child nutrition"[tiab] OR "energy intake"[tiab] OR "feeding behavior*"[tiab] OR "feeding practice*"[tiab] OR "diet practice*"[tiab] OR "dietary practice*"[tiab] OR "pediatric nutrition"[tiab] OR nutrition[tiab] OR "infant nutrition"[tiab] OR "child nutrition"[tiab] OR consumption[tiab] OR "food consumption"[tiab] OR "diet, food, and nutrition"[tiab]) AND (2000:2021[pdat]) AND (english[Filter])

Additional databases searched: **Agricola, CABI**, EbscoHost’s **Academic Search Premier** and **EconLit**.

### Search strategy for question 3

(("Livestock"[Mesh] OR "Poultry"[Mesh] OR "Sheep, Domestic"[Mesh] OR "Buffaloes"[Mesh] OR "Reindeer"[Mesh] OR "Goats"[Mesh] OR "Columbiformes"[Mesh] OR "Anseriformes"[Mesh] OR "Galliformes"[Mesh] OR "Chickens"[Mesh] OR "Turkeys"[Mesh] OR "Camelidae"[Mesh] OR "Rabbits"[Mesh] OR "Horses"[Mesh] OR "Guinea Pigs"[Mesh] OR "farm animal*"[tiab] OR "domestic animal*"[tiab] OR "domesticated animal*"[tiab] OR livestock[tiab] OR horse*[tiab] OR donkey*[tiab] OR cattle[tiab] OR cow[tiab] OR cows[tiab] OR ox[tiab] OR oxen[tiab] OR zebu*[tiab] OR bali*[tiab] OR yak[tiab] OR yaks[tiab] OR "water buffalo*"[tiab] OR gayal*[tiab] OR sheep*[tiab] OR goat*[tiab] OR reindeer*[tiab] OR pig[tiab] OR pigs[tiab] OR hog[tiab] OR hogs[tiab] OR swine[tiab] OR chicken*[tiab] OR duck*[tiab] OR poultr*[tiab] OR geese[tiab] OR goose[tiab] OR buffalo*[tiab] OR "domestic fowl*"[tiab] OR fowl*[tiab] OR camel*[tiab] OR llama*[tiab] OR alpaca*[tiab] OR rabbit*[tiab] OR "guinea pig*"[tiab] OR turkey*[tiab] OR pigeon*[tiab] OR porcine[tiab] OR bovine*[tiab] OR mule*[tiab] OR foal*[tiab] OR calf[tiab] OR calves[tiab] OR gosling*[tiab] OR duckling*[tiab] OR piglet*[tiab]) **AND** ("Animal Husbandry"[Mesh] OR smallhold*[tiab] OR "small hold*"[tiab] OR "small-hold*"[tiab] OR "small-scale farm*"[tiab] OR "small scale farm*"[tiab] OR "small-scale agriculture*"[tiab] OR "small scale agriculture*"[tiab] OR subsistence[tiab] OR "subsistence farm*"[tiab] OR "subsistence farming"[tiab] OR "family farm"[tiab] OR "pastoral"[tiab] OR "herding"[tiab] OR "agro-pastoral"[tiab] OR "agropastoral"[tiab] OR "agro pastoral"[tiab])) **AND** ("Empowerment"[Mesh] OR "Decision Making, Shared"[Mesh] OR "Gender Role"[Mesh] OR "Personal Autonomy"[Mesh] OR "Interpersonal Relations"[Mesh] OR "Gender Equity"[Mesh] OR "Women's Rights"[Mesh] OR "women's empowerment"[tiab] OR empower*[tiab] OR "woman's income"[tiab] OR "women's income"[tiab] OR "women's status"[tiab] OR "woman's status"[tiab] OR "decision-making"[tiab] OR "decision making"[tiab] OR "bargaining power"[tiab] OR power[tiab] OR wealth[tiab] OR "extra-domestic"[tiab] OR "gender parity"[tiab] OR "gender equality"[tiab] OR "labor force participation"[tiab] OR "economic participation"[tiab] OR "inclusive development"[tiab] OR autonomy[tiab] OR "women's power"[tiab] OR "women's advancement"[tiab] OR "productive potential"[tiab] OR "male privilege*"[tiab] OR "access to income"[tiab] OR "contribution to household* welfare"[tiab] OR agency[tiab] OR "women's agency"[tiab] OR "shared decision making"[tiab] OR "gender role"[tiab] OR "personal autonomy"[tiab] OR "interpersonal relation*"[tiab] OR "gender equity"[tiab] OR "women's right*"[tiab] OR "woman's control"[tiab] OR "women's control"[tiab] OR "woman's participation"[tiab] OR "women's participation"[tiab]) AND (2000:2021[pdat]) AND (english[Filter])

Additional databases searched: **CABI** and EbscoHost’s **Academic Search Premier, EconLit** and **Women’s Studies International**.

## Sample (PubMed) search strategies for questions studying risks of smallholder livestock production

### Search strategy for question 1

#### Search strategy for enteric pathogens that are associated with environmental enteric dysfunction (EED): EED

("Sprue, Tropical/epidemiology"[Mesh] OR "Sprue, Tropical/microbiology"[Mesh] OR "Sprue, Tropical/parasitology"[Mesh] OR "Sprue, Tropical/pathology"[Mesh] OR "Sprue, Tropical/physiology"[Mesh] OR "Sprue, Tropical/physiopathology"[Mesh] OR "Sprue, Tropical/prevention and control"[Mesh] OR "environmental enteropath*"[tiab] OR "environmental enteric dysfunction*"[tiab] OR "tropical enteropath*"[tiab] OR "pediatric environmental enteropath*"[tiab]) AND (2000:2021[pdat]) AND (english[Filter])

Additional databases searched**: Embase, Web of Science Core Collection** and **CABI (Global Health)**.

#### Search strategy for enteric pathogens that are associated with undernutrition outcomes: (Enteric pathogens) AND (infant and children) AND (undernutrition outcomes)

("Gastrointestinal Diseases/epidemiology"[Mesh] OR "Gastrointestinal Diseases/etiology"[Mesh] OR "Gastrointestinal Diseases/microbiology"[Mesh] OR "Gastrointestinal Diseases/parasitology"[Mesh] OR "Gastrointestinal Diseases/prevention and control"[Mesh] OR "Gastrointestinal Diseases/transmission"[Mesh] OR "Gastrointestinal Diseases/virology"[Mesh] OR "Escherichia coli Infections/epidemiology"[Mesh] OR "Escherichia coli Infections/microbiology"[Mesh] OR "Escherichia coli Infections/prevention and control"[Mesh] OR "Escherichia coli Infections/transmission"[Mesh] ) OR "Campylobacter"[Mesh] OR "Cryptosporidium"[Mesh] OR "Giardia"[Mesh] OR "Adenoviridae"[Mesh] OR "Aeromonas"[Mesh] OR "Ascaris"[Mesh] OR "Astroviridae"[Mesh] OR "Cyclospora"[Mesh] OR "Hymenolepis nana"[Mesh] OR "Ancylostomatoidea"[Mesh] OR "Isospora"[Mesh] OR "Norovirus"[Mesh] OR "Plesiomonas"[Mesh] OR "Rotavirus"[Mesh] OR "Salmonella"[Mesh] OR "Sapovirus"[Mesh] OR "Shigella"[Mesh] OR "Strongyloides"[Mesh] OR "Trichuris"[Mesh] OR "Vibrio cholerae"[Mesh] OR "Yersinia"[Mesh] OR "enteric pathogen*"[tiab] OR enteropathogen*[tiab] OR "entero pathogen*"[tiab] OR "entero-pathogen*"[tiab] OR enterobacteria*[tiab] OR "entero bacteria*"[tiab] OR "entero-bacteria*"[tiab] OR "enteric bacteria"[tiab] OR "enteric protozoa*"[tiab] OR "enteric virus"[tiab] OR enterovir*[tiab] OR "entero vir*"[tiab] OR "entero-vir*"[tiab] OR (diarrheagenic[tiab] OR "diarrhea-genic"[tiab] OR "diarrhea genic"[tiab] OR enteroaggregative[tiab] OR "entero aggregative"[tiab] OR "entero-aggregative"[tiab] OR enterohemorrhagic[tiab] OR "entero hemorrhagic"[tiab] OR "entero-hemorrhagic"[tiab] OR enteroinvasive[tiab] OR "entero invasive"[tiab] OR "entero-invasive"[tiab] OR enteropathogenic[tiab] OR "entero pathogenic"[tiab] OR "entero-pathogenic"[tiab] OR enterotoxigenic[tiab] OR "entero toxigenic"[tiab] OR "entero-toxigenic"[tiab]) AND ("E. coli"[tiab] OR "Escherichia coli"[tiab] OR "EAEC"[tiab] OR "Enteroaggregative Escherichia coli"[tiab] OR "EPEC"[tiab] OR"ETEC"[tiab] OR "EHEC"[tiab] OR "EIEC"[tiab] OR "Enteroinvasive Escherichia coli"[tiab] OR campylobacter*[tiab] OR cryptosporidi*[tiab] OR giardia*[tiab] OR adenovirus[tiab] OR aeromonas[tiab] OR ascaris[tiab] OR astrovir*[tiab] OR cyclospor*[tiab] OR hymenolepi*[tiab] OR histolytica[tiab] OR hookworm*[tiab] OR ancylostoma*[tiab] OR necator*[tiab] OR isospora[tiab] OR norovirus[tiab] OR plesiomonas[tiab] OR rotavirus[tiab] OR salmonell*[tiab] OR sapovirus[tiab] OR shiga*[tiab] OR "STEC"[tiab] OR shigell*[tiab] OR strongyloides[tiab] OR trichuri*[tiab] OR "vibrio cholerae"[tiab] OR yersini*[tiab]) **AND** ("Infant"[Mesh] OR "Child, Preschool"[Mesh] OR infant*[tiab] OR children[tiab]) **AND** (“Growth Disorders/epidemiology”[Mesh] OR stunt*[tiab] OR "linear growth"[tiab] OR wast*[tiab] OR underweight[tiab])Filters: English, from 2000 – 2021

Additional databases searched**: Embase, Web of Science Core Collection** and **CABI (Global Health)**.

### Search strategy for question 2

**Search strategy: (livestock OR feces) AND (enteric pathogens) AND (smallholder)**

(“Livestock"[Mesh] OR "Poultry"[Mesh] OR "Sheep, Domestic"[Mesh] OR "Buffaloes"[Mesh] OR "Reindeer"[Mesh] OR "Goats"[Mesh] OR "Columbiformes"[Mesh] OR "Anseriformes"[Mesh] OR "Galliformes"[Mesh] OR "Chickens"[Mesh] OR "Turkeys"[Mesh] OR "Camelidae"[Mesh] OR "Rabbits"[Mesh] OR "Horses"[Mesh] OR "Guinea Pigs"[Mesh] OR (("Animals, Suckling"[Mesh] OR "Animals, Newborn"[Mesh]) AND ("Animals, Domestic"[Mesh])) OR ( "Disease Reservoirs/epidemiology"[Mesh] OR "Disease Reservoirs/microbiology"[Mesh] OR "Disease Reservoirs/parasitology"[Mesh] OR "Disease Reservoirs/prevention and control"[Mesh] OR "Disease Reservoirs/virology"[Mesh] OR "animal reservoir*"[tiab] OR "farm animal*"[tiab] OR "domestic animal*"[tiab] OR "domesticated animal*"[tiab] OR livestock[tiab] OR horse*[tiab] OR donkey*[tiab] OR cattle[tiab] OR cow[tiab] OR cows[tiab] OR ox[tiab] OR oxen[tiab] OR zebu*[tiab] OR bali*[tiab] OR yak[tiab] OR yaks[tiab] OR "water buffalo*"[tiab] OR gayal*[tiab] OR sheep*[tiab] OR goat*[tiab] OR reindeer*[tiab] OR pig[tiab] OR pigs[tiab] OR hog[tiab] OR hogs[tiab] OR swine[tiab] OR chicken*[tiab] OR duck*[tiab] OR poultr*[tiab] OR geese[tiab] OR goose[tiab] OR buffalo*[tiab] OR "domestic fowl*"[tiab] OR fowl*[tiab] OR camel*[tiab] OR llama*[tiab] OR alpaca*[tiab] OR rabbit*[tiab] OR "guinea pig*"[tiab] OR turkey*[tiab] OR pigeon*[tiab] OR porcine[tiab] OR bovine*[tiab] OR mule*[tiab] OR foal*[tiab] OR calf[tiab] OR calves[tiab] OR gosling*[tiab] OR duckling*[tiab] OR piglet*[tiab]) OR ("Feces/epidemiology"[Mesh] OR "Feces/microbiology"[Mesh] OR "Feces/parasitology"[Mesh] OR "Feces/prevention and control"[Mesh] OR "Feces/virology"[Mesh]) OR ("Manure/microbiology"[Mesh] OR "Manure/parasitology"[Mesh] OR "Manure/virology"[Mesh] OR feces[tiab] OR faeces[tiab] OR fecal[tiab] OR faecal[tiab] OR "animal waste"[tiab] OR manure[tiab] OR dung[tiab] OR dropping*[tiab]) **AND** (("enteric pathogen*"[tiab] OR enteropathogen*[tiab] OR "entero pathogen*"[tiab] OR "entero-pathogen*"[tiab] OR enterobacteria*[tiab] OR "entero bacteria*"[tiab] OR "entero-bacteria*"[tiab] OR "enteric bacteria"[tiab] OR "enteric protozoa*"[tiab] OR "enteric virus"[tiab] OR enterovir*[tiab] OR "entero vir*"[tiab] OR "entero-vir*"[tiab] OR ((diarrheagenic[tiab] OR "diarrhea-genic"[tiab] OR "diarrhea genic"[tiab] OR enteroaggregative[tiab] OR "entero aggregative"[tiab] OR "entero-aggregative"[tiab] OR enterohemorrhagic[tiab] OR "entero hemorrhagic"[tiab] OR "entero-hemorrhagic"[tiab] OR enteroinvasive[tiab] OR "entero invasive"[tiab] OR "entero-invasive"[tiab] OR enteropathogenic[tiab] OR "entero pathogenic"[tiab] OR "entero-pathogenic"[tiab] OR enterotoxigenic[tiab] OR "entero toxigenic"[tiab] OR "entero-toxigenic"[tiab]) **AND** ("Gastrointestinal Diseases/epidemiology"[Mesh] OR "Gastrointestinal Diseases/etiology"[Mesh] OR "Gastrointestinal Diseases/microbiology"[Mesh] OR "Gastrointestinal Diseases/parasitology"[Mesh] OR "Gastrointestinal Diseases/prevention and control"[Mesh] OR "Gastrointestinal Diseases/transmission"[Mesh] OR "Gastrointestinal Diseases/virology"[Mesh] OR "Escherichia coli Infections/epidemiology"[Mesh] OR "Escherichia coli Infections/microbiology"[Mesh] OR "Escherichia coli Infections/prevention and control"[Mesh] OR "Escherichia coli Infections/transmission"[Mesh] ) OR "Campylobacter"[Mesh] OR "Cryptosporidium"[Mesh] OR "Giardia"[Mesh] OR "Adenoviridae"[Mesh] OR "Aeromonas"[Mesh] OR "Ascaris"[Mesh] OR "Astroviridae"[Mesh] OR "Cyclospora"[Mesh] OR "Hymenolepis nana"[Mesh] OR "Ancylostomatoidea"[Mesh] OR "Isospora"[Mesh] OR "Norovirus"[Mesh] OR "Plesiomonas"[Mesh] OR "Rotavirus"[Mesh] OR "Salmonella"[Mesh] OR "Sapovirus"[Mesh] OR "Shigella"[Mesh] OR "Strongyloides"[Mesh] OR "Trichuris"[Mesh] OR "Vibrio cholerae"[Mesh] OR "Yersinia"[Mesh] OR "E. coli"[tiab] OR "Escherichia coli"[tiab] OR "EAEC"[tiab] OR "Enteroaggregative Escherichia coli"[tiab] OR "EPEC"[tiab] OR"ETEC"[tiab] OR "EHEC"[tiab] OR "EIEC"[tiab] OR "Enteroinvasive Escherichia coli"[tiab] OR campylobacter*[tiab] OR cryptosporidi*[tiab] OR giardia*[tiab] OR adenovirus[tiab] OR aeromonas[tiab] OR ascaris[tiab] OR astrovir*[tiab] OR cyclospor*[tiab] OR hymenolepi*[tiab] OR histolytica[tiab] OR hookworm*[tiab] OR ancylostoma*[tiab] OR necator*[tiab] OR isospora[tiab] OR norovirus[tiab] OR plesiomonas[tiab] OR rotavirus[tiab] OR salmonell*[tiab] OR sapovirus[tiab] OR shiga*[tiab] OR "STEC"[tiab] OR shigell*[tiab] OR strongyloides[tiab] OR trichuri*[tiab] OR "vibrio cholerae"[tiab] OR yersini*[tiab]) **AND** (Animal Husbandry"[Mesh] OR "Rural Population"[Mesh] OR smallhold*[tiab] OR "small hold*"[tiab] OR "small-hold*"[tiab] OR "small-scale farm*"[tiab] OR "smallscale farm*"[tiab] OR "small scale farm*"[tiab] OR "small-scale agriculture*"[tiab] OR "smallscale agriculture*"[tiab] OR "small scale agriculture*"[tiab] OR subsistence[tiab] OR "subsistence farm*"[tiab] OR "subsistence farming"[tiab] OR "family farm"[tiab] OR "pastoral"[tiab] OR "herding"[tiab] OR "agro-pastoral"[tiab] OR "agropastoral"[tiab] OR "agro pastoral"[tiab]) AND (2000:2021[pdat]) AND (english[Filter])

Additional databases searched**: Embase, Web of Science Core Collection** and **CABI (Global Health)**.

### Search strategy for question 3

#### Source attribution technologies: (Source attribution) AND (enteric pathogens) AND (Animal/food/drinking water)

("source-track*"[tiab] OR "source track*"[tiab] OR "source attribut*"[tiab] OR "source-attribut*"[tiab]) **AND** ("Gastrointestinal Diseases/epidemiology"[Mesh] OR "Gastrointestinal Diseases/etiology"[Mesh] OR "Gastrointestinal Diseases/microbiology"[Mesh] OR "Gastrointestinal Diseases/parasitology"[Mesh] OR "Gastrointestinal Diseases/prevention and control"[Mesh] OR "Gastrointestinal Diseases/transmission"[Mesh] OR "Gastrointestinal Diseases/virology"[Mesh] OR "Escherichia coli Infections/epidemiology"[Mesh] OR "Escherichia coli Infections/microbiology"[Mesh] OR "Escherichia coli Infections/prevention and control"[Mesh] OR "Escherichia coli Infections/transmission"[Mesh] OR "Campylobacter"[Mesh] OR "Cryptosporidium"[Mesh] OR "Giardia"[Mesh] OR "Adenoviridae"[Mesh] OR "Aeromonas"[Mesh] OR "Ascaris"[Mesh] OR "Astroviridae"[Mesh] OR "Cyclospora"[Mesh] OR "Hymenolepis nana"[Mesh] OR "Ancylostomatoidea"[Mesh] OR "Isospora"[Mesh] OR "Norovirus"[Mesh] OR "Plesiomonas"[Mesh] OR "Rotavirus"[Mesh] OR "Salmonella"[Mesh] OR "Sapovirus"[Mesh] OR "Shigella"[Mesh] OR "Strongyloides"[Mesh] OR "Trichuris"[Mesh] OR "Vibrio cholerae"[Mesh] OR "Yersinia"[Mesh] OR "E. coli"[tiab] OR "Escherichia coli"[tiab] OR "EAEC"[tiab] OR "Enteroaggregative Escherichia coli"[tiab] OR "EPEC"[tiab] OR"ETEC"[tiab] OR "EHEC"[tiab] OR "EIEC"[tiab] OR “Enteroinvasive Escherichia coli”[tiab] OR campylobacter*[tiab] OR cryptosporidi*[tiab] OR giardia*[tiab] OR adenovirus[tiab] OR aeromonas[tiab] OR ascaris[tiab] OR astrovir*[tiab] OR cyclospor*[tiab] OR hymenolepi*[tiab] OR histolytica[tiab] OR hookworm*[tiab] OR ancylostoma*[tiab] OR necator*[tiab] OR isospora[tiab] OR norovirus[tiab] OR plesiomonas[tiab] OR rotavirus[tiab] OR salmonell*[tiab] OR sapovirus[tiab] OR shiga*[tiab] OR "STEC"[tiab] OR shigell*[tiab] OR strongyloides[tiab] OR trichuri*[tiab] OR "vibrio cholerae"[tiab] OR yersini*[tiab] OR "enteric pathogen*"[tiab] OR enteropathogen*[tiab] OR “entero pathogen*”[tiab] OR “entero-pathogen*”[tiab] OR enterobacteria*[tiab] OR “entero bacteria*”[tiab] OR “entero-bacteria*”[tiab] OR "enteric bacteria"[tiab] OR "enteric protozoa*"[tiab] OR "enteric virus"[tiab] OR enterovir*[tiab] OR “entero vir*”[tiab] OR “entero-vir*”[tiab] OR diarrheagenic[tiab] OR “diarrhea-genic”[tiab] OR “diarrhea genic”[tiab] OR enteroaggregative[tiab] OR “entero aggregative”[tiab] OR “entero-aggregative”[tiab] OR enterohemorrhagic[tiab] OR “entero hemorrhagic”[tiab] OR “entero-hemorrhagic”[tiab] OR enteroinvasive[tiab] OR “entero invasive”[tiab] OR “entero-invasive”[tiab] OR enteropathogenic[tiab] OR “entero pathogenic”[tiab] OR “entero-pathogenic”[tiab] OR enterotoxigenic[tiab] OR “entero toxigenic”[tiab] OR “entero-toxigenic”[tiab]) **AND** ("Livestock"[Mesh] OR "Poultry"[Mesh] OR "Sheep, Domestic"[Mesh] OR "Buffaloes"[Mesh] OR "Reindeer"[Mesh] OR "Goats"[Mesh] OR "Columbiformes"[Mesh] OR "Anseriformes"[Mesh] OR "Galliformes"[Mesh] OR "Chickens"[Mesh] OR "Turkeys"[Mesh] OR "Camelidae"[Mesh] OR "Rabbits"[Mesh] OR "Horses"[Mesh] OR "Guinea Pigs"[Mesh] OR "Animals, Suckling"[Mesh] OR "Animals, Newborn"[Mesh] OR "Animals, Domestic"[Mesh] OR "farm animal*"[tiab] OR "domestic animal*"[tiab] OR “domesticated animal*”[tiab] OR livestock[tiab] OR horse*[tiab] OR donkey*[tiab] OR cattle[tiab] OR cow[tiab] OR cows[tiab] OR ox[tiab] OR oxen[tiab] OR zebu*[tiab] OR bali*[tiab] OR yak[tiab] OR yaks[tiab] OR "water buffalo*"[tiab] OR gayal*[tiab] OR sheep*[tiab] OR goat*[tiab] OR reindeer*[tiab] OR pig[tiab] OR pigs[tiab] OR hog[tiab] OR hogs[tiab] OR swine[tiab] OR chicken*[tiab] OR duck*[tiab] OR poultr*[tiab] OR geese[tiab] OR goose[tiab] OR buffalo*[tiab] OR “domestic fowl*”[tiab] OR fowl*[tiab] OR camel*[tiab] OR llama*[tiab] OR alpaca*[tiab] OR rabbit*[tiab] OR "guinea pig*"[tiab] OR turkey*[tiab] OR pigeon*[tiab] OR porcine[tiab] OR bovine*[tiab] OR mule*[tiab] OR foal*[tiab] OR calf[tiab] OR calves[tiab] OR gosling*[tiab] OR duckling*[tiab] OR piglet*[tiab]) **AND** ("Disease Reservoirs/epidemiology"[Mesh] OR "Disease Reservoirs/microbiology"[Mesh] OR "Disease Reservoirs/parasitology"[Mesh] OR "Disease Reservoirs/prevention and control"[Mesh] OR "Disease Reservoirs/virology"[Mesh] OR "Food/epidemiology"[Mesh] OR "Food/microbiology"[Mesh] OR "Food/parasitology"[Mesh] OR "Food/virology"[Mesh] OR "Drinking Water/microbiology"[Mesh] OR "Drinking Water/parasitology"[Mesh] OR "Drinking Water/virology"[Mesh] OR "animal reservoir*"[tiab]) AND (2000:2021[pdat]) AND (english[Filter])

Additional databases searched**: Embase, Web of Science Core Collection** and **CABI (Global Health)**.

#### Control methods

##### Animal waste management

((waste*[tiab] OR manure[tiab] OR dung[tiab] OR feces[tiab] OR faeces[tiab] OR fecal[tiab] OR faecal[tiab]) **AND** ("Livestock"[Mesh] OR "Poultry"[Mesh] OR animal[tiab] OR livestock[tiab] OR poultr*[tiab] OR cow [tiab] OR cows [tiab] OR pig [tiab] OR pigs [tiab] OR swine* [tiab] OR hog [tiab] OR hogs [tiab] OR goat* [tiab] OR sheep* [tiab] OR lamb* [tiab] OR chicken* [tiab] OR goose [tiab] OR geese [tiab] OR duck [tiab] OR ducks [tiab] OR ducking* [tiab] OR piglet* [tiab] OR calf [tiab] OR calves [tiab] OR buffalo* [tiab])) **AND** (("Waste management"[MeSH] OR manag*[tiab] OR treat*[tiab] OR compost*[tiab] OR "bio-digester*"[tiab] OR "bio digester*"[tiab] OR biodigester*[tiab] OR "vegetative buffer*"[tiab] OR "riparian buffer*"[tiab] OR "anaerobic digest*"[tiab] OR "vegetative filter strip*"[tiab] OR "vegetated filter strip*"[tiab]) **OR** (reduc*[tiab] OR inactiv*[tiab])**) AND** ("low-income countr*" OR "low income countr*" OR "middle-income countr*" OR "middle income countr*" OR "low- and middle-income countr*" OR "Afghanistan"[Mesh] OR "Benin"[Mesh] OR "Burkina Faso"[Mesh] OR "Burundi"[Mesh] OR "Cambodia"[Mesh] OR "Central African Republic"[Mesh] OR "Chad"[Mesh] OR "Comoros"[Mesh] OR "Democratic People's Republic of Korea"[Mesh] OR "Democratic Republic of the Congo"[Mesh] OR "Ethiopia"[Mesh] OR "Eritrea"[Mesh] OR "Gambia"[Mesh] OR "Guinea"[Mesh] OR "Guinea-Bissau"[Mesh] OR "Haiti"[Mesh] OR "Liberia"[Mesh] OR "Madagascar"[Mesh] OR "Malawi"[Mesh] OR "Mali"[Mesh] OR "Mozambique"[Mesh] OR "Nepal"[Mesh] OR "Niger"[Mesh] OR "Rwanda"[Mesh] OR "Sierra Leone"[Mesh] OR "Somalia"[Mesh] OR "South Sudan"[Mesh] OR "Tanzania"[Mesh] OR "Togo"[Mesh] OR "Uganda"[Mesh] OR "Zimbabwe"[Mesh] "Armenia"[Mesh] OR "Bangladesh"[Mesh] OR "Bhutan"[Mesh] OR "Bolivia"[Mesh] OR "Cameroon"[Mesh] OR "Cabo Verde"[Mesh] OR "Congo"[Mesh] OR "Cote d'Ivoire"[Mesh] OR "Djibouti"[Mesh] OR "Egypt"[Mesh] OR "El Salvador"[Mesh] OR "Georgia (Republic)"[Mesh] OR "Ghana"[Mesh] OR "Guatemala"[Mesh] OR "Guyana"[Mesh] OR "Honduras"[Mesh] OR "India"[Mesh] OR "Indonesia"[Mesh] OR "Kenya"[Mesh] OR "Kosovo"[Mesh] OR "Kyrgyzstan"[Mesh] OR "Laos"[Mesh] OR "Lesotho"[Mesh] OR "Mauritania"[Mesh] OR "Micronesia"[Mesh] OR "Moldova"[Mesh] OR "Morocco"[Mesh] OR "Myanmar"[Mesh] OR "Nicaragua"[Mesh] OR "Nigeria"[Mesh] OR "Pakistan"[Mesh] OR "Papua New Guinea"[Mesh] OR "Philippines"[Mesh] OR "Independent State of Samoa"[Mesh] OR "Senegal"[Mesh] OR "Sri Lanka"[Mesh] OR "Eswatini"[Mesh] OR "Syria"[Mesh] OR "Tajikistan"[Mesh] OR "Timor-Leste"[Mesh] OR "Ukraine"[Mesh] OR "Uzbekistan"[Mesh] OR "Vanuatu"[Mesh] OR "Vietnam"[Mesh] OR "Yemen"[Mesh] OR "Zambia"[Mesh]) AND ((english[Filter]) AND (2000:2021[pdat]))

Additional databases searched**: Embase, Web of Science Core Collection** and **CABI (Global Health)**.

##### Animal movement containment

(("Livestock"[Mesh] OR "Poultry"[Mesh] OR cow [tiab] OR cows [tiab] OR pig [tiab] OR pigs [tiab] OR swine* [tiab] OR hog [tiab] OR hogs [tiab] OR goat* [tiab] OR sheep* [tiab] OR lamb* [tiab] OR chicken* [tiab] OR goose [tiab] OR geese [tiab] OR duck [tiab] OR ducks [tiab] OR ducking* [tiab] OR piglet* [tiab] OR calf [tiab] OR calves [tiab] OR buffalo* [tiab]) **AND** ("Feces/adverse effects"[Mesh] OR "Feces/microbiology"[Mesh] OR "Feces/parasitology"[Mesh] OR "animal waste"[tiab] OR feces [tiab] OR faeces [tiab] OR faecal [tiab] OR fecal [tiab] OR dropping* [tiab] OR dung*[tiab] OR "enteric pathogen*"[tiab])) OR ("child growth" OR stunting OR EED OR diarrhea OR diarrhoea) **AND** (corral*[tiab] OR pen[tiab] OR pens[tiab] OR "animal movement"[tiab]) **AND** (("small scale"[tiab] OR "small-scale"[tiab] OR family[tiab]) **AND** (farm*[tiab] OR agriculture[tiab] OR husbandry[tiab])) AND ((english[Filter]) AND (2000:2021[pdat]))

Additional databases searched**: Embase, Web of Science Core Collection** and **CABI (Global Health)**.

##### Milking hygiene/milk treatment

(("Hygiene"[Mesh] OR "Sanitation"[Mesh] OR milking[tiab]) **AND** (hygiene[tiab] OR sanita*[tiab])) OR (("Milk"[Mesh] OR milk[tiab] OR dairy[tiab] OR "dairy product*"[tiab] OR "milk product*"[tiab] OR yogurt*[tiab] OR yoghurt[tiab] OR "Yogurt"[Mesh]) **AND** (pasteuris*[tiab] OR pasteuriz*[tiab] OR boil*[tiab])) **AND** ("small scale"[tiab] OR "small-scale"[tiab] OR smallhold*[tiab] OR "small hold*"[tiab] OR household*[tiab]) AND (english[Filter]) AND (2000:2021[pdat])

Additional databases searched**: Embase, Web of Science Core Collection** and **CABI (Global Health)**.

##### Slaughtering hygiene

(contaminat*[tiab] OR decontaminat*[tiab] OR hygiene[tiab] OR hygienic[tiab]) **AND** (carcass*[tiab] OR slaughter*[tiab] OR butcher*[tiab]) **AND** ("small scale"[tiab] OR "small-scale"[tiab] OR smallhold*[tiab] OR "small hold*"[tiab]) AND ((english[Filter]) AND (2000:2021[pdat]))

Additional databases searched**: Embase, Web of Science Core Collection** and **CABI (Global Health)**.

##### Food preparation and fomites

("Disinfection"[Mesh] OR "Detergents"[Mesh] OR disinfect*[tiab] OR "Detergents"[Mesh] OR detergent*[tiab] OR wash*[tiab] OR clean*[tiab] OR soap*[tiab]) **AND** ("Cooking and Eating Utensils"[Mesh] OR kitchen[tiab] OR "food preparation"[tiab] OR "eating utensils"[tiab] OR surfac*[tiab] OR dish*[tiab] OR bowl*[tiab] OR plate*[tiab] OR "cutting board"[tiab] OR "butcher block"[tiab] OR cutter*[tiab] OR blade*[tiab] OR mincer*[tiab] OR bottle*[tiab] OR floors[tiab] OR "Play and Playthings"[Mesh] OR toy[tiab] OR toys[tiab] OR "household fomite*"[tiab]) **AND** (("Enteritis/prevention and control"[Mesh] OR "Food Contamination/prevention and control"[Mesh] OR "Diarrhea/prevention and control"[Mesh] OR "enteric pathogen*"[tiab] OR "food contaminat*"[tiab] OR diarrhea[tiab] OR diarrhoea[tiab] OR enteritis[tiab]) **AND** (prevent*[tiab] OR control*[tiab] OR manag*[tiab] OR interven*[tiab])) **AND** ("low-income countr*" OR "middle-income countr*" OR "low- and middle-income countr*" OR "low income countr*" OR "middle income countr*" OR Afghanistan OR Afghan* OR Benin* OR "Burkina Faso" OR Burundi* OR Cambodia* OR "Central African Republic" OR Chad OR Comoros OR "Democratic Republic of the Congo" OR Eritrea* OR Ethiopia* OR Gambia* OR Guinea* OR Haiti* OR "North Korea" OR "North Korean" OR Liberia* OR Madagascar OR malagas* OR Malawi* OR Mali* OR Mozambiqu* OR Nepal* OR Niger* OR Rwanda* OR "Sierra Leon*" OR Somalia* OR "South Sudan*" OR Tanzania* OR Togo* OR Uganda* OR Zimbabwe* OR Armenia* OR Bangladesh* OR Bhutan* OR Bolivia* OR Cameroon* OR "Cape Verde" OR "Republic of the Congo" OR "Cote d’Ivoire" OR Djibouti* OR "Arab Republic of Egypt" or Egypt* OR "El Salvador" OR Salvadoran* OR Georgia* OR Ghana* OR Guatemala* OR Guyana* OR Honduras OR Honduran* OR India OR Indonesia* OR Kenya* OR Kiribati* OR Kosovo OR Kosovan* OR Kyrgyz* OR "Kyrgyz Republic" OR "Lao People’s Democratic Republic" OR Laos OR Laotian* OR Lesotho* OR Mauritania* OR Micronesia* OR Moldova* OR Morocc* OR Myanmar OR Burma OR Burmese OR Nicaragua* OR Nigeria* OR Pakistan* OR "Papua New Guinea" OR "new guinean*" OR Philippines OR Filpin* OR Samoa* OR "Sao Tome and Principe" OR Senegal* OR "Solomon Islands" OR "Sri Lanka" OR "sri lankan" OR "sri lankans" OR Ceylon* OR Sudan* OR Swaziland OR Syria* OR Tajikistan* OR "Timor-Leste" OR Ukraine OR Ukrainian* OR Uzbekistan* OR Vanuatu* OR Vietnam* OR "Viet Nam" OR Vietnam* OR Gaza* OR "West Bank" OR Yemen* OR Zambia* OR Albania* OR Algeria* OR "American Samoa" OR Angola* OR Azerbaijan* OR Belarus* OR Belize* OR "Bosnia and Herzegovina" OR Bosnian* OR Botswana* OR Brazil* OR Bulgaria* OR China OR Chinese OR "Costa Rica" OR Cuba* OR Dominica* OR "Dominican Republic" OR Ecuador* OR Fiji* OR Gabon* OR Grenada* OR "Islamic Republic of Iran" OR Iran* OR Iraq* OR Jamaica* OR Jordan* OR Kazakhstan* OR Lebanon OR Lebanese OR Libya* OR Macedonia* OR Malaysia* OR Maldiv* OR "Marshall Island*" OR Mauritius OR Mauritian* OR Mexic* OR Mongolia* OR Montenegro OR Montenegrin* OR Namibia* OR Palau* OR Panama OR Panamanian* OR Paraguay* OR Peru* OR Romania* OR Serbia* OR "South Africa*" OR "St Lucia*" OR "St. Vincent and the Grenadines" OR "St Vincent" OR "Grenadines" OR Surinam* OR Thai* OR Tonga* OR Tunisia* OR Turkmenistan* OR Tuvalu*) AND ((english[Filter]) AND (2000:2021[pdat]))

Additional databases searched**: Embase, Web of Science Core Collection** and **CABI (Global Health)**.

##### Food preservation

("Food Preservation"[Mesh] OR ferment*[tiab] OR "air-dry"[tiab] OR "air dry"[tiab] OR "air dried" [tiab] OR "air-dried"[tiab]) **AND** (milk[tiab] OR dairy[tiab] OR yogurt[tiab] OR yoghurt[tiab] OR meat*[tiab] OR egg[tiab] OR eggs[tiab]) **AND** ("smallhold*"[tiab] OR "small hold*"[tiab] OR "small-hold*"[tiab] OR "small-scale"[tiab] OR "smallscale"[tiab] OR "household*"[tiab]) AND ((english[Filter]) AND (2000:2021[pdat]))

Additional databases searched**: Embase, Web of Science Core Collection** and **CABI (Global Health)**.

##### Food and water storage

("Food Storage"[Mesh] OR "food storage"[tiab] OR “stored water”[tiab] OR "Cooking and Eating Utensils"[Mesh] OR "eating utensil*"[tiab] OR "cooking utensil*"[tiab] OR knife[tiab] OR knives[tiab] OR fork*[tiab] OR spoon*[tiab] OR pan[tiab] OR pans[tiab] OR pot[tiab] OR pots[tiab] OR bowl*[tiab] OR plate*[tiab]) **AND (**("Hygiene"[Mesh] OR hygiene[tiab] OR "Enteritis/prevention and control"[Mesh] OR "Food Contamination/prevention and control"[Mesh] OR "Diarrhea/prevention and control"[Mesh]) OR (("food contaminat*"[tiab] OR "enteric pathogen*"[tiab] OR diarrhea[tiab] OR diarrhoea[tiab] OR enteritis[tiab]) **AND** (prevent*[tiab] OR control*[tiab] OR manag*[tiab] OR interven*[tiab]))) **AND** ("low-income countr*" OR "middle-income countr*" OR "low- and middle-income countr*" OR "low income countr*" OR "middle income countr*" OR Afghanistan OR Afghan* OR Benin* OR "Burkina Faso" OR Burundi* OR Cambodia* OR "Central African Republic" OR Chad OR Comoros OR "Democratic Republic of the Congo" OR Eritrea* OR Ethiopia* OR Gambia* OR Guinea* OR Haiti* OR "North Korea" OR "North Korean" OR Liberia* OR Madagascar OR malagas* OR Malawi* OR Mali* OR Mozambiqu* OR Nepal* OR Niger* OR Rwanda* OR "Sierra Leon*" OR Somalia* OR "South Sudan*" OR Tanzania* OR Togo* OR Uganda* OR Zimbabwe* OR Armenia* OR Bangladesh* OR Bhutan* OR Bolivia* OR Cameroon* OR "Cape Verde" OR "Republic of the Congo" OR "Cote d’Ivoire" OR Djibouti* OR "Arab Republic of Egypt" or Egypt* OR "El Salvador" OR Salvadoran* OR Georgia* OR Ghana* OR Guatemala* OR Guyana* OR Honduras OR Honduran* OR India OR Indonesia* OR Kenya* OR Kiribati* OR Kosovo OR Kosovan* OR Kyrgyz* OR "Kyrgyz Republic" OR "Lao People’s Democratic Republic" OR Laos OR Laotian* OR Lesotho* OR Mauritania* OR Micronesia* OR Moldova* OR Morocc* OR Myanmar OR Burma OR Burmese OR Nicaragua* OR Nigeria* OR Pakistan* OR "Papua New Guinea" OR "new guinean*" OR Philippines OR Filpin* OR Samoa* OR "Sao Tome and Principe" OR Senegal* OR "Solomon Islands" OR "Sri Lanka" OR "sri lankan" OR "sri lankans" OR Ceylon* OR Sudan* OR Swaziland OR Syria* OR Tajikistan* OR "Timor-Leste" OR Ukraine OR Ukrainian* OR Uzbekistan* OR Vanuatu* OR Vietnam* OR "Viet Nam" OR Vietnam* OR Gaza* OR "West Bank" OR Yemen* OR Zambia* OR Albania* OR Algeria* OR "American Samoa" OR Angola* OR Azerbaijan* OR Belarus* OR Belize* OR "Bosnia and Herzegovina" OR Bosnian* OR Botswana* OR Brazil* OR Bulgaria* OR China OR Chinese OR "Costa Rica" OR Cuba* OR Dominica* OR "Dominican Republic" OR Ecuador* OR Fiji* OR Gabon* OR Grenada* OR "Islamic Republic of Iran" OR Iran* OR Iraq* OR Jamaica* OR Jordan* OR Kazakhstan* OR Lebanon OR Lebanese OR Libya* OR Macedonia* OR Malaysia* OR Maldiv* OR "Marshall Island*" OR Mauritius OR Mauritian* OR Mexic* OR Mongolia* OR Montenegro OR Montenegrin* OR Namibia* OR Palau* OR Panama OR Panamanian* OR Paraguay* OR Peru* OR Romania* OR Serbia* OR "South Africa*" OR "St Lucia*" OR "St. Vincent and the Grenadines" OR "St Vincent" OR "Grenadines" OR Surinam* OR Thai* OR Tonga* OR Tunisia* OR Turkmenistan* OR Tuvalu*) AND ((english[Filter]) AND (2000:2021[pdat]))

Additional databases searched**: Embase, Web of Science Core Collection** and **CABI (Global Health)**.

##### Food and feeding hygiene

("Hand Hygiene"[Mesh] OR "hand hygiene*"[tiab] OR hygiene [tiab] OR "hand-washing"[tiab] OR "hand washing"[tiab] OR "handwash*"[tiab] OR boil*[tiab] OR reheat*[tiab]) **OR** ("Food Handling" [Mesh] OR "food prepar*"[tiab] OR "food handl*"[tiab] OR "Bottle Feeding"[Mesh] OR "bottle feed*"[tiab] OR "bottle fed"[tiab] OR "baby bottle*"[tiab] OR "feeding bottle*"[tiab] OR "Feeding Behavior"[Mesh] OR "feeding behaviour*"[tiab] OR "feeding behavior*"[tiab] OR "eating behaviour*"[tiab] OR "eating behavior*"[tiab]) **AND** ("Enteritis/prevention and control"[Mesh] OR "Food Contamination/prevention and control"[Mesh] OR "Diarrhea/prevention and control"[Mesh]) **AND** ("low-income countr*" OR "middle-income countr*" OR "low- and middle-income countr*" OR "low income countr*" OR "middle income countr*" OR Afghanistan OR Afghan* OR Benin* OR "Burkina Faso" OR Burundi* OR Cambodia* OR "Central African Republic" OR Chad OR Comoros OR "Democratic Republic of the Congo" OR Eritrea* OR Ethiopia* OR Gambia* OR Guinea* OR Haiti* OR "North Korea" OR "North Korean" OR Liberia* OR Madagascar OR malagas* OR Malawi* OR Mali* OR Mozambiqu* OR Nepal* OR Niger* OR Rwanda* OR "Sierra Leon*" OR Somalia* OR "South Sudan*" OR Tanzania* OR Togo* OR Uganda* OR Zimbabwe* OR Armenia* OR Bangladesh* OR Bhutan* OR Bolivia* OR Cameroon* OR "Cape Verde" OR "Republic of the Congo" OR "Cote d’Ivoire" OR Djibouti* OR "Arab Republic of Egypt" or Egypt* OR "El Salvador" OR Salvadoran* OR Georgia* OR Ghana* OR Guatemala* OR Guyana* OR Honduras OR Honduran* OR India OR Indonesia* OR Kenya* OR Kiribati* OR Kosovo OR Kosovan* OR Kyrgyz* OR "Kyrgyz Republic" OR "Lao People’s Democratic Republic" OR Laos OR Laotian* OR Lesotho* OR Mauritania* OR Micronesia* OR Moldova* OR Morocc* OR Myanmar OR Burma OR Burmese OR Nicaragua* OR Nigeria* OR Pakistan* OR "Papua New Guinea" OR "new guinean*" OR Philippines OR Filpin* OR Samoa* OR "Sao Tome and Principe" OR Senegal* OR "Solomon Islands" OR "Sri Lanka" OR "sri lankan" OR "sri lankans" OR Ceylon* OR Sudan* OR Swaziland OR Syria* OR Tajikistan* OR "Timor-Leste" OR Ukraine OR Ukrainian* OR Uzbekistan* OR Vanuatu* OR Vietnam* OR "Viet Nam" OR Vietnam* OR Gaza* OR "West Bank" OR Yemen* OR Zambia* OR Albania* OR Algeria* OR "American Samoa" OR Angola* OR Azerbaijan* OR Belarus* OR Belize* OR "Bosnia and Herzegovina" OR Bosnian* OR Botswana* OR Brazil* OR Bulgaria* OR China OR Chinese OR "Costa Rica" OR Cuba* OR Dominica* OR "Dominican Republic" OR Ecuador* OR Fiji* OR Gabon* OR Grenada* OR "Islamic Republic of Iran" OR Iran* OR Iraq* OR Jamaica* OR Jordan* OR Kazakhstan* OR Lebanon OR Lebanese OR Libya* OR Macedonia* OR Malaysia* OR Maldiv* OR "Marshall Island*" OR Mauritius OR Mauritian* OR Mexic* OR Mongolia* OR Montenegro OR Montenegrin* OR Namibia* OR Palau* OR Panama OR Panamanian* OR Paraguay* OR Peru* OR Romania* OR Serbia* OR "South Africa*" OR "St Lucia*" OR "St. Vincent and the Grenadines" OR "St Vincent" OR "Grenadines" OR Surinam* OR Thai* OR Tonga* OR Tunisia* OR Turkmenistan* OR Tuvalu*) AND ((english[Filter]) AND (2000:2021[pdat])

Additional databases searched**: Embase, Web of Science Core Collection** and **CABI (Global Health)**.

# Supplementary information 2

## Eligibility criteria for literature studying the benefits of smallholder livestock production

### Eligibility criteria for literature studying question 1

**Inclusion Criteria:**

- Publication Types: Any
- Languages of Full-text: English
- Publication Dates: 2000 onward
- Populations Studied: Infants and young children (0-5 years) living in smallholder OR pastoralist families/groups
- Geographic Areas: Low- and middle- income countries (LMIC)

**Exclusion Criteria:**

- Articles that do not meet one or more of the above inclusion criteria
- Publication Types: No letters, editorials, comments unless they comment on weaknesses in studies that may/will be included in the present study; No dissertations, meeting abstracts, proceedings papers
- Articles for which full-text was not readily available at our institution
- Articles that do not investigate household or child diet

### Eligibility criteria for literature studying question 2

**Inclusion Criteria:**

- Publication Types: Any
- Languages of Full-text: English
- Publication Dates: 2000 onward
- Populations Studied: Infants and young children (0-5 years) living in smallholder OR pastoralist families/groups
- Geographic Areas: Low- and middle- income countries (LMIC)

**Exclusion Criteria:**

- Articles that do not meet one or more of the above inclusion criteria
- Publication Types: No letters, editorials, comments unless they comment on weaknesses in studies that may/will be included in the present study; No dissertations, meeting abstracts, proceedings papers
- Articles for which full-text was not readily available at our institution
- Articles that do not investigate household or child diet and household income, sale, or expenditure

### Eligibility criteria for literature studying question 3

**Inclusion Criteria:**

- Publication Types: Any
- Languages of Full-text: English
- Publication Dates: 2000 onward
- Populations Studied: Infants and young children (0-5 years) living in smallholder OR pastoralist families/groups
- Geographic Areas: Low- and middle- income countries (LMIC)

**Exclusion Criteria:**

- Articles that do not meet one or more of the above inclusion criteria
- Publication Types: No letters, editorials, comments unless they comment on weaknesses in studies that may/will be included in the present study; No dissertations, meeting abstracts, proceedings papers
- Articles for which full-text was not readily available at our institution
- Articles that do not investigate household or child diet and women’s empowerment

## Eligibility criteria for literature studying the risks of smallholder livestock production

### Eligibility criteria for literature studying question 1

#### Eligibility criteria for literature studying pathogens associated with environmental enteric dysfunction (EED)

**Inclusion Criteria:**

- Publication Types: Any
- Languages of Full-text: English
- Publication Dates: 2000 onward
- Populations Studied: Infants and young children (0-5 years) OR experimental animals
- Geographic Areas: Low- and middle- income countries (LMIC)/low-resource settings in upper middle-income countries, or laboratories conducting animal studies

**Exclusion Criteria:**

- Articles that do not meet one or more of the above inclusion criteria
- Articles that do not mention pathogens related to poor gut health, or related risk factors and pathways, or reservoirs of those pathogens
- Publication Types: No letters, editorials, comments unless they comment on weaknesses in studies that may/will be included in the present study; No dissertations, meeting abstracts, proceedings papers
- Articles for which full-text was not readily available at our institution

#### Eligibility criteria for literature studying pathogens associated with undernutrition

**Inclusion Criteria:**

- Publication Types: Any
- Languages of Full-text: English
- Publication Dates: 2000 onward
- Populations studied: Infants and young children (0-5 years)
- Geographic areas: Low- and middle- income countries (LMIC)/low-resource settings in upper middle-income countries
- **Exclusion Criteria:**
- Articles that do not meet one or more of the above inclusion criteria
- Articles that do not mention pathogens that are statistically and significantly associated with stunting/linear growth faltering/wasting/underweight
- Publication Types: No letters, editorials, comments unless they comment on weaknesses in studies that may/will be included in the present study; No dissertations, meeting abstracts, proceedings papers
- Articles for which full-text was not readily available at our institution

### Eligibility criteria for literature studying question 2

**Inclusion Criteria:**

- Publication Types: Any
- Languages of Full-text: English
- Publication Dates: 2000 onward
- Populations studied: Households living in smallholder OR pastoralist families/groups (including neighbors)
- Geographic areas: Low- and middle- income countries (LMIC)/low-resource settings in upper middle-income countries

**Exclusion Criteria:**

- Articles that do not meet one or more of the above inclusion criteria
- Animal: Studies focusing on "wild" animals or birds (not housed/fed regularly), fish, reptiles, amphibians or insect; studies without involvement of livestock (e.g., WaSH-related studies in settings without livestock production); livestock farming settings in western style intensive husbandry
- Publication Types: No letters, editorials, comments unless they comment on weaknesses in studies that may/will be included in the present study; No dissertations; conference proceedings and meeting abstracts; book chapters
- Studies focus on only non-enteric pathogens
- Articles only study antibiotic resistance, speciation, or prevalence of pathogen in livestock
- Exclusion criteria included articles for which full-text was not readily available at our institution

### Eligibility criteria for literature studying question 3

#### Eligibility criteria for literature studying source attribution technologies

**Inclusion Criteria:**

- Publication Types: Any
- Languages of Full-text: English
- Publication Dates: 2000 onward
- Populations studied: All
- Geographic areas: Low- and middle- income countries (LMIC)/low-resource settings in upper middle-income countries

**Exclusion Criteria:**

- Articles that do not meet one or more of the above inclusion criteria
- Articles that do not mention pathogens affecting children’s gut health, fecal indicator bacteria with a specific animal origin, or related source of human exposure [i.e., livestock (no wildlife or aquatic life), food, drinking water (no marine, river/creek, ground, recreational water or natural watershed)]
- Articles do not mention source-tracking/attribution technologies
- Publication Types: No letters, editorials, comments unless they comment on weaknesses in studies that may/will be included in the present study; No dissertations, meeting abstracts, proceedings papers
- Exclusion criteria included articles for which full-text was not readily available at our institution

#### Eligibility criteria for literature studying control methods

**Inclusion Criteria:**

- Publication Types: Any
- Languages of Full-text: English
- Publication Dates: 2000 onward
- Populations studied: All
- Geographic areas: Low- and middle- income countries (LMIC)/low-resource settings in upper middle-income countries

**Exclusion Criteria:**

- Articles that do not meet one or more of the above inclusion criteria
- Articles that do not include interventions carried out on study subjects
- Publication Types: No letters, editorials, comments unless they comment on weaknesses in studies that may/will be included in the present study; No dissertations, meeting abstracts, proceedings papers
- Exclusion criteria included articles for which full-text was not readily available at our institution

# Supplementary information 3

## Quantitative summary of search results and screening process

| Theme | Question (specification) | Items identified through database searches, personal libraries and following references | Duplicates removed through Covidence | Unique items screened* (TIAB level) | # FT screened | # included |
| --- | --- | --- | --- | --- | --- | --- |
| Benefits of smallholder livestock production | 1, 2, and 3^*^ | 3517 | 703 | 2814 | 555 | 145 |
| Risks of smallholder livestock production | 1 (EED-associated pathogens) | 1125 | 640 | 485 | 166 | 12 |
|  | 1 (undernutrition-associated pathogens) | 1228 | 541 | 687 | 91 | 38 |
|  | 2 | 4971 | 1411 | 3560 | 218 | 27 |
|  | 3 (attribution technologies) | 950 | 622 | 328 | 131 | 15 |
|  | 3 (Control methods) | 5851 | 2586 | 3265 | 66 | 29 |

^*^One search strategy was used in each database (including one for the Ebsco combined-databases search) to retrieve literature on all three questions
